# Fluorescent supramolecular polymers of barbiturate dyes with thiophene-cored twisted π-systems†

**DOI:** 10.1039/d1sc06246h

**Published:** 2021-12-09

**Authors:** Maika Kawaura, Takumi Aizawa, Sho Takahashi, Hiroshi Miyasaka, Hikaru Sotome, Shiki Yagai

**Affiliations:** Division of Advanced Science and Engineering, Graduate School of Science and Engineering, Chiba University 1-33 Yayoi-cho, Inage-ku Chiba 263-8522 Japan; Division of Frontier Materials Science, Graduate School of Engineering Science, Osaka University 1-3 Machikaneyama, Toyonaka Osaka 560-8531 Japan sotome@laser.chem.es.osaka-u.ac.jp; Department of Applied Chemistry and Biotechnology, Graduate School of Engineering, Chiba University 1-33 Yayoi-cho, Inage-ku Chiba 263-8522 Japan yagai@faculty.chiba-u.jp; Institute for Global Prominent Research (IGPR), Chiba University 1-33 Yayoi-cho, Inage-ku Chiba 263-8522 Japan

## Abstract

Because supramolecular polymerization of emissive π-conjugated molecules depends strongly on π–π stacking interaction, the formation of well-defined one-dimensional nanostructures often results in a decrease or only a small increase of emission efficiency. This is also true for our barbiturate-based supramolecular polymers wherein hydrogen-bonded rosettes of barbiturates stack quasi-one-dimensionally through π–π stacking interaction. Herein we report supramolecular polymerization-induced emission of two regioisomeric 2,3-diphenylthiophene derivatives functionalized with barbituric acid and tri(dodecyloxy)benzyl wedge units. In CHCl_3_, both compounds are molecularly dissolved and accordingly poorly emissive due to a torsion-induced non-radiative decay. In methylcyclohexane-rich conditions, these barbiturates self-assemble to form crystalline nanofibers and exhibit strongly enhanced emission through supramolecular polymerization driven by hydrogen-bonding. Our structural analysis suggests that the barbiturates form a tape-like hydrogen-bonding motif, which is rationalized by considering that the twisted geometries of 2,3-diphenylthiophene cores prevend the competing rosettes from stacking into columnar supramolecular polymers. We also found that a small difference in the molecular polarity originating from the substitutional position of the thiophene core influences interchain association of the supramolecular polymers, affording different luminescent soft materials, gel and nanosheet.

## Abstract 2

Two barbiturate dyes with regioisomeric thiophene-cored twisted π-systems show strongly enhanced emission through supramolecular polymerization. The supramolecular polymers thus formed exhibit distinct emission colors and degree of agglomeration.

## Graphical abstract

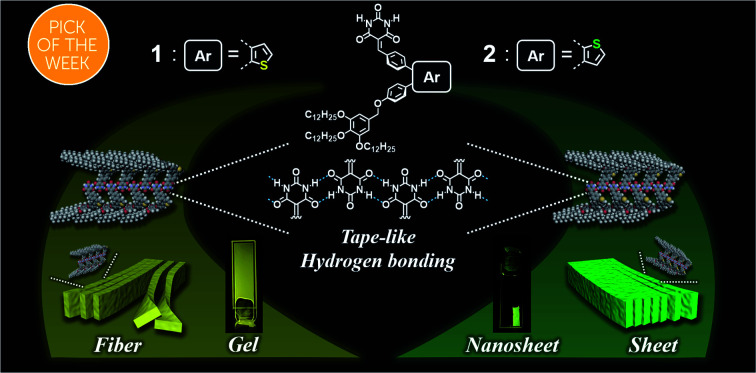

## Introduction

Design of purely organic luminescent materials is important to develop cost-effective organic devices such as organic light-emitting diodes (OLEDs)^1^ and organic light-emitting transistors (OLETs).^2^ One of the dilemmas that organic materials researchers often face is emission quenching in the solid state due to strong electronic interactions among molecules.^3^ This dilemma becomes more pronounced when one aims to give organic molecules the ability to self-assemble into specific nanostructures that are advantageous for device fabrication, because such an ability relies on specific intermolecular interaction. Thus, sterically crowded molecules have been designed so far to limit undesirable interactions in the aggregated state, and they have been investigated for various applications as innovative materials showing aggregation-induced emission (AIE).^4^ However, in terms of the supramolecular self-assembly field, “aggregation” of these molecules has still room to be engineered supramolecularly to obtain well-defined nanostructures like nanofibers and nanosheets.^5^

**Fig. 1:**
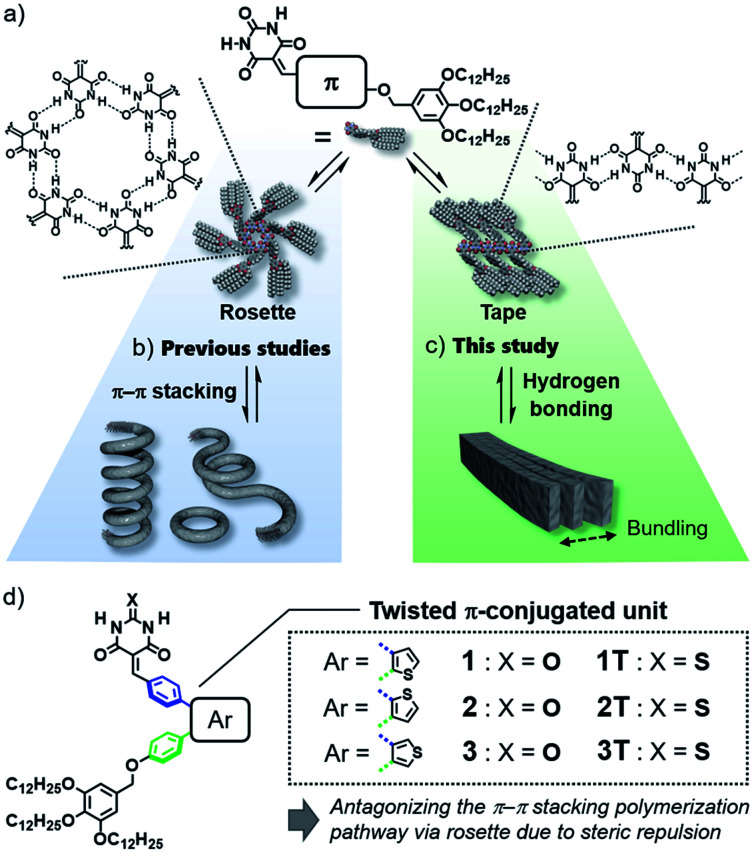
(a) Rosette (left) and tape-like hydrogen-bonded motifs (right) of π-conjugated barbiturates. (b) and (c) Schematic diagram of hierarchical self-assembly of π-conjugated barbiturates *via* (b) rosette and (c) tape pathways. (d) Chemical structures of compounds 1–3 and reference compounds 1T–3T.

In the above context, even classical supramolecular units are attractive to design aggregation-induced emission luminogens (AIEgens) that can organize into well-defined one-dimensional structures.^6^ As one such supramolecular unit, we are interested in barbiturate,^7^ which have long been known to exhibit crystalline polymorph by the formation of infinite tape-like hydrogen-bonded motifs (Fig. 1a, right).^7*a*^ Barbiturates conjugated with aromatic units have been known to be emissive in crystalline state although they are non-emissive in solution because of torsion-induced non-radiative decay.^8^ They have also been used by several researchers as a (self-)complementary hydrogen-bonding supramolecular unit.^9^ In particular, the formation of cyclic hexamers (rosettes, Fig. 1a, left) by barbiturates functionalized with an aromatic unit and bulky solubilizing chains has been found by our group, and the formation of topological supramolecular polymers by the rosettes equipped with π-conjugated units illustrates the importance of higher-order structures of one-dimensional nanomaterials to control their properties (Fig. 1b).^10^ However, the luminescence efficiency of these soft supramolecular polymers is at most 10% due to the excimer formation of π-conjugated units, which act as the primary interactive cites for the polymerization. In the present study, we addressed one-dimensionally nanostructured luminescent materials by utilizing direct supramolecular polymerization of barbiturates through crystalline tape-like hydrogen-bonding motif.

In the hydrogen-bond-directed self-assembly of our π-conjugated barbiturates, the formation of rosette (cyclization) and tape (polymerization) are competing under the situation wherein aromatic units are well-solvated.^11*b*^ Because rosettes are discrete species, they can nucleate toward supramolecular polymerization through π–π stacking,^11^ and accordingly competing tape-like species cannot be obtained (Fig. 1b). In this context, the introduction of sterically demanding aromatic systems would suppress the nucleation of rosettes, and open the pathway leading to tape-like species (Fig. 1c). The bulky AIEgens are a unit that reasonably meets this requirement, and hierarchical organization of the resulting tape-like supramolecular polymers can show AIE property. We thus designed and synthesized barbiturates 1–3 conjugated with twisted diphenylthiophene (DPT) units (Fig. 1d). These three regioisomeric barbiturates differ in the substituted position of the central thiophene ring. We revealed that barbiturates 1 and 2 show strong emission due to the formation of supramolecular polymers formed *via* a tape-like hydrogen-bonded motif. The resulting supramolecular polymers of 1 and 2 not only exhibited different emission colors but also formed different nanostructures reflecting a small difference in the polarity of the molecules. We further investigated the excited state dynamics of the aggregates with different morphologies by ultrafast laser spectroscopy.

## Results and discussion

### Photophysical properties

**Fig. 2:**
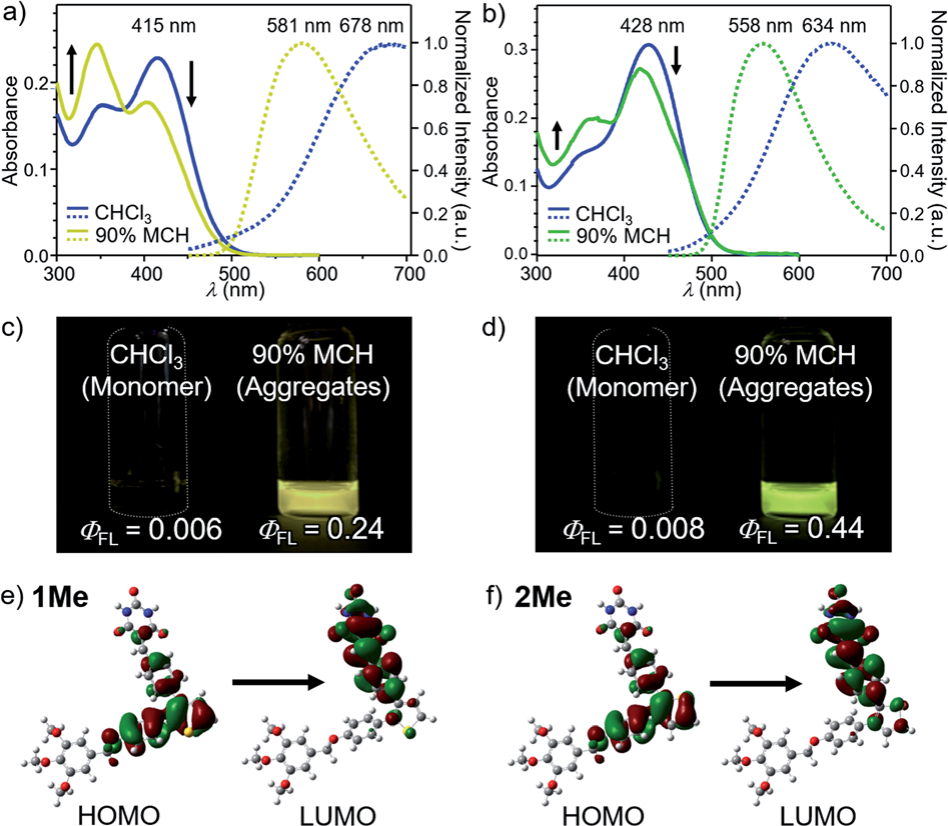
(a) and (b) UV/Vis absorption (left axis) and fluorescence spectra (right axis, excited at 377 nm for 1 and 392 nm for 2) of (a) 1 and (b) 2 in MCH/CHCl_3_ mixture (MCH fraction = 0% and 90%) (*c* = 100 μM). (c) and (d) Photographs of (c) 1 and (d) 2 in CHCl_3_ (left) and 90 : 10 MCH/CHCl_3_ (v/v) mixture (right) under 365 nm light illumination (*c* = 100 μM). (e) and (f) Molecular orbitals of the S_0_ → S_1_ transition for model compounds (e) 1Me and (f) 2Me (see ESI†) calculated at the TD-CAM-B3LYP/6-31+G(d,p) level of theory. The molecular structures of 1Me and 2Me were optimized by DFT calculation at the CAM-B3LYP/6-31+G(d,p) level of theory. For all calculations, dodecyl groups were replaced with methyl groups.

Absorption spectra of molecularly dispersed 1 and 2 in CHCl_3_ (100 μM) show absorption maxima at 415 and 428 nm (Fig. 2a and b). Upon exciting these bands, both compounds are poorly emissive (*Φ*_FL_ < 0.01) due to a torsion-induced non-radiative decay (Fig. 2c and d), as represented by large rate constants of non-radiative decay (Table S1†). The emission maxima of 1 and 2 were recorded at *λ*_max_ ≈ 678 nm and *λ*_max_ = 634 nm, respectively. The larger Stokes shift of 1 than 2 (1: 9347 cm^−1^, 2: 7592 cm^−1^) can be qualitatively reproduced by time-dependent density functional theory (TD-DFT) calculations (CAM-B3LYP/6-31+G(d,p) level) using model compounds (Fig. S1†), and indicates that 1 undergoes the larger torsional relaxation in the excited state. The calculation also revealed that the corresponding absorption and fluorescence bands are mainly attributed to the intramolecular charge-transfer (CT) transition between diphenylthiophene (donor, HOMO) and benzylidene barbiturate moieties (acceptor, LUMO) (Fig. 2e and f). This characterization indicates that the CT character of the excited state also contributes to the large Stokes shifts.

Because 1 and 2 were very soluble in CHCl_3_ but scarcely soluble in methylcyclohexane (MCH), we prepared assemblies by injecting their CHCl_3_ solution (100 μL) into stirred MCH (900 μL) (*c* = 100 μM). In 90 : 10 MCH/CHCl_3_ (v/v) solvent mixture, these compounds became emissive, and showed a fluorescence band at *λ*_max_ = 581 nm for 1 and at *λ*_max_ = 558 nm for 2 (Fig. 2a–d). The fluorescence quantum yields (*Φ*_FL_) were evaluated as 0.24 for 1 and 0.44 for 2, respectively. These values are significantly higher than those of the previously reported supramolecular polymers based on hydrogen-bonded rosettes (*Φ*_FL_ ≈ 0.1), implying the emergence of a different self-assembly species.

In accordance with the fluorescence enhancement, appreciable changes were observed for the UV/Vis absorption spectra of 1 and 2 in CHCl_3_ and 90 : 10 MCH/CHCl_3_ mixture. In CHCl_3_, both dyes displayed two absorption bands with different intensity ratios (Fig. 2a and b). In 90 : 10 MCH/CHCl_3_ mixture, the intensity of the longer-wavelength bands attenuated concomitantly with a slight blue shift while that of the shorter-wavelength ones intensified. This change is obviously pronounced for 1 as the intensity of the two bands were reversed. Although this pronounced change of the absorption spectra of 1 is a typical signature of non-emissive H-type aggregates, the aggregated 1 and 2 in the present study are both highly emissive and maintain radiative rate constants comparable to the molecularly dispersive state, which clearly excludes the exciton coupling upon simple H-type aggregation (Table S1†).^12^ As another explanation for the absorption spectral change, one may quote distinct vibrational signature of absorption bands observed for J- and H-type aggregation of dyes, which cannot be explained by conventional exciton theory.^13^ However, the two absorption bands of 1 and 2 are not vibrational transition but rather ascribable to charge-transfer transition according to the TD-DFT study. We thus considered another possibility as follows.

To reveal the origin of the spectral change lying behind the supramolecular aggregation, we performed TD-DFT calculations for model compounds 1Me and 2Me having methyl groups in place of dodecyl groups. The relevant absorption bands were simulated as a function of intramolecular structural degrees of freedom. Upon decreasing the dihedral angle between the thiophene ring and the benzyloxy-substituted phenylene ring in the donor moiety, the longer and shorter wavelength bands complementarily decreased and increased, respectively (Fig. S2†). This reproduces the observed change of the absorption intensities upon aggregation, while dependences on the dihedral motions of other moieties were not in line with experimental one (Fig. S3 and S4†). These results suggest that the absorption spectral changes are mainly due to the planarization of the DPT units upon the stacking of the adjacent molecules in aggregation. This interpretation is consistent with the smaller Stokes shift of the aggregates (1: 7540 cm^−1^, 2: 6034 cm^−1^) than the molecularly dispersive state, that is, the torsional motion is suppressed in the rigid environment of the aggregated state.

### Nanostructures

**Fig. 3:**
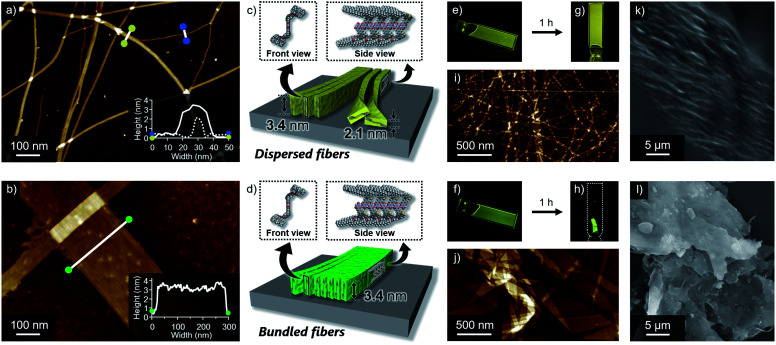
(a) and (b) AFM images of (a) 1 and (b) 2 aggregates formed in 90 : 10 MCH/CHCl_3_ mixture (*c* = 100 μM). Insets show cross-section analysis between the yellow dots (solid line), blue dots (dash line) and green dots in the image of (a) and (b), respectively. (c) and (d) Schematic representation of tape-like supramolecular fibers of (c) 1 and (d) 2 with different bundling tendency. (e) and (f) Photographs of as-prepared viscous liquids of (e) 1 and (f) 2 in 90 : 10 MCH/CHCl_3_ mixture under 365 nm light illumination (*c* = 3 mM). (g) and (h) Photographs of (g) gel of 1 and (h) precipitates of 2 under 365 nm light illumination (*c* = 3 mM). (i) and (j) AFM images of (i) dried gel of 1 and (j) precipitates of 2. (k) and (l) SEM images of (k) dried gel of 1 and (l) precipitates of 2.

**Fig. 4:**
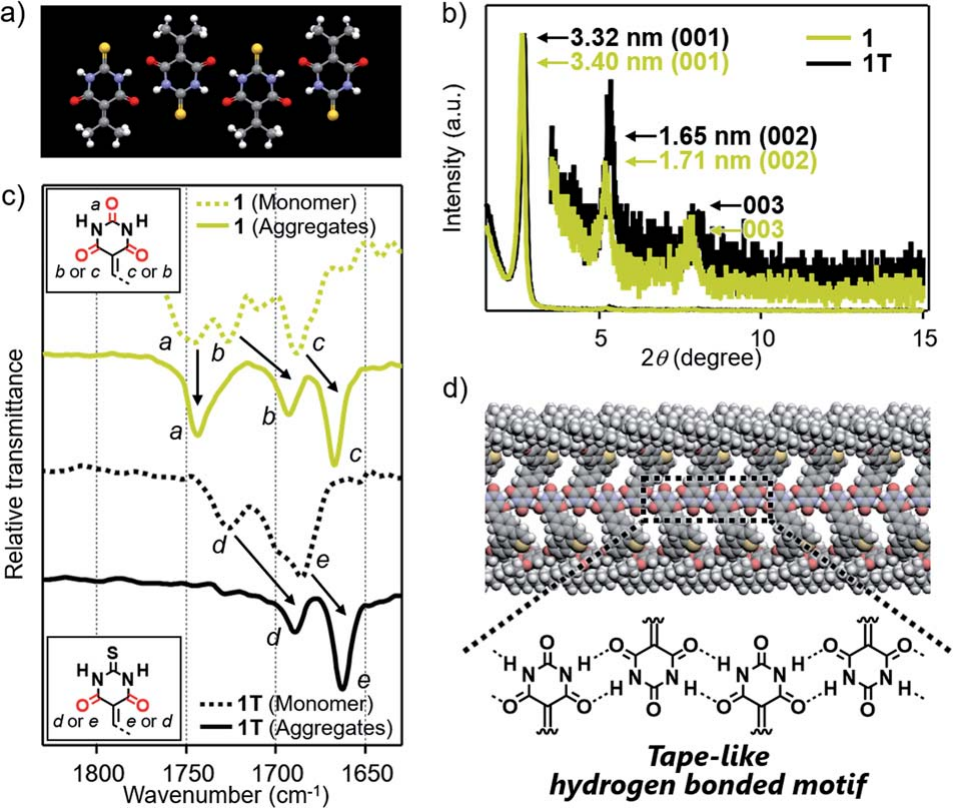
(a) X-ray crystal structures of 5-(isopropylidene)-2-thiobarbituric acid (CCDC 989216) along hydrogen-bonding direction.^16^ (b) Powder XRD pattern of bulk sample of 1 and 1T, which are obtained by aging each of these molecules in 90 : 10 MCH/CHCl_3_ mixture at room temperature (*c* = 100 μM). (c) FT-IR spectra (C=O stretching region) of 1 and 1T in monomeric (CHCl_3_) or aggregated state (90 : 10 MCH/CHCl_3_ mixture) (*c* = 100 μM). (d) Schematic illustration of the proposed hydrogen-bonded motif of 1.

Self-assembled morphologies of 1 and 2 were investigated by atomic force microscopy (AFM). AFM imaging of the assemblies, spin-coated from the above luminescent solutions onto highly oriented pyrolytic graphite (HOPG), visualized linearly extended fibers for both dyes, but with clearly different degrees of two-dimensional aggregation (Fig. 3a and b). Compared to well-dispersed fibers of 1, those of 2 are heavily bundled to form two-dimensional sheet-like structures. The *z*-axis height of both fibers are 3.4 nm, indicating that they are based on the same supramolecular packing (Fig. 3c and d). This notion was supported by the almost same 3.4 nm spacing lamellar X-ray diffraction (XRD) patterns of the precipitated fibers obtained by aging the solutions (Fig. 4b and S5†). Importantly, for less bundled fibers of 1, a closer observation of the AFM images revealed elementary fibers with markedly smaller thickness of 2.1 nm (Fig. 3a). These elementary fibers are branched from the bundled fibers with 3.4 nm height. The observation indicates that the elementary fibers correspond to single supramolecular polymer chains having height of 3.4 nm and thickness of 2.1 nm, which were adsorbed to the HOPG substrate in the face-on arrangement. In reference to molecular modelling, we propose supramolecular polymerization of 1 and 2 based on a tape-like hydrogen-bonded array of barbituric acid units (Fig. 3c and d).

The observed difference in two-dimensional aggregation (bundling) tendency of 1 and 2 can be reasonably rationalized by the larger dipole moment of 2 than 1 as suggested from the DFT calculation (Fig. S6†). The calculation showed that the ground-state dipole moment of 2 (5.15 D) is slightly larger than that of 1 (4.37 D). Such a difference, albeit small in the molecular level, could affect macroscopic properties of the orderly assembled structures. Namely, more polar fibers of 2 should have stronger cohesive force in the nonpolar medium, and be able to bundle smoothly.^14^ In fact, a further two-dimensional organization of the fibers of 1 was also observed upon aging the solution for 15 hours without stirring (Fig. S7†). Because this aged solution of 1 did not show further increase of fluorescence quantum yield, the different emission efficiencies of aggregated 1 and 2 do not correlate with the two-dimensional aggregation tendency of supramolecular fibers. The influence of dipole moment on the two-dimensional aggregation was further supported by another regioisomer 3 with almost equal dipole moment (4.28 D) to that of 1. This regioisomer resulted in the formation of supramolecular fibers, which are well-dispersed comparably to those of 1 under the same condition (Fig. S8†). It is worthy to note that only slightly enhanced emission was observed for 3 upon changing solvent from CHCl_3_ (*Φ*_FL_ = 0.003) to 90 : 10 MCH/CHCl_3_ mixture (*Φ*_FL_ = 0.04). For this regioisomer, sufficient suppression of intramolecular motion cannot be expected upon aggregation because the single-like C–C bond of the thiophene ring connecting the two phenylene moieties could increase intrinsic conformational flexibility of the entire π-conjugated system.^15^

Reflecting obvious difference in the two-dimensional aggregation tendency of supramolecular fibers of 1 and 2, their formation at a higher monomer concentration resulted in different soft materials. When 1 and 2 were dissolved in 90 : 10 MCH/CHCl_3_ mixture at *c* = 3 mM by vigorous heating and the resulting homogeneous solutions were naturally cooled to room temperature, both solutions immediately became viscous (Fig. 3e and f). Upon standing them at room temperature for 1 h, gelation was observed for 1, whereas a phase-separated thin film which kept the shape of the 1 mm cuvette was obtained from 2 (Fig. 3g and h). AFM and SEM imaging after drying these materials showed the formation of entangled fibers and sheet-like structures for 1 and 2, respectively, in nano-to-micrometer hierarchical scales (Fig. 3i–l).

### Hydrogen-bonding motifs

Based on the above structural studies, we propose that 1 and 2 self-assemble by a polymeric hydrogen-bonding pattern of the barbiturate unit to give tape-like supramolecular polymers. Since it was difficult to obtain a single crystal suitable for X-ray structure analysis either from 1 or 2, we studied the corresponding 2-thiobarbiturate derivatives 1T and 2T, respectively (Fig. 1d) to predict the hydrogen bonding pattern of 1 and 2 among the hydrogen-bonded polymorphs of barbiturates. Due to the weaker hydrogen-bond acceptor character of C=S group, 2-thiobarbituric acid derivatives have been reported to form hydrogen bonding motif using only C=O groups, as shown in Fig. 4a.^7*a*,16^ Despite this defect on hydrogen-bonding ability, AFM and XRD studied demonstrated that 1T and 2T can form supramolecular fibers with almost identical structural and bundling features with those of 1 and 2 under the same condition (Fig. 4b, S5 and S9†). Moreover, Fourier transform infrared (FT-IR) spectra of 1 and 2 in CHCl_3_ (monomeric state) and in 90 : 10 MCH/CHCl_3_ mixture (aggregated state) exhibited large lower-wavenumber shifts of C=O vibrational bands due to hydrogen-bonding only for two C=O groups, while the band of the C(2)=O group did not show such a large shift (Fig. 4c and S10†). Almost the same observation was noted for the IR studies of 1T and 2T (Fig. 4c and S10†). Combined with the AFM and XRD results, we propose supramolecular polymerization of 1 and 2 driven by hydrogen-bonding using two NH groups and C(4)=O and C(6)=O groups (Fig. 4d).

### Excited state dynamics

**Fig. 5:**
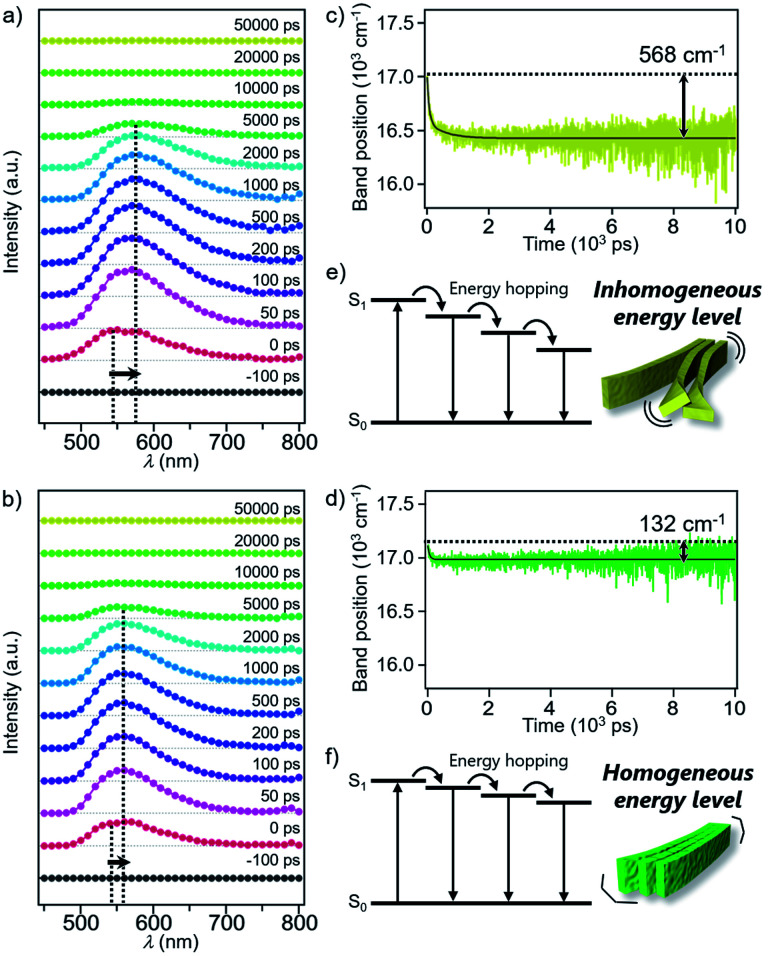
(a) and (b) Time-resolved fluorescence spectra of the aggregates of (a) 1 and (b) 2. 1 and 2 were excited at 430 nm. (c) and (d) Time evolutions of the fluorescence band position (center of mass) of (c) 1 and (d) 2. Black line is bi- or mono-exponential fit. (e) and (f) Schematic diagram of energy hopping in the aggregates of (e) 1 and (f) 2 at the molecular level.

Excited state dynamics of 1 and 2 depending on different morphologies were studied by time-resolved fluorescence measurements of the monomeric and aggregated states.^17^ Upon excitation of CHCl_3_ solutions of 1 and 2 at 430 nm, fluorescence bands appeared immediately after excitation and underwent dynamic red-shifts within 100 ps (Fig. S11†). This dynamic red-shift is ascribable to the relaxation of monomeric 1 and 2 in the excited state caused by torsional motion and solvent reorientation. On the other hand, fluorescence bands of aggregated 1 and 2 in 90 : 10 MCH/CHCl_3_ mixture decayed in several hundreds of ps with smaller red-shifts than that of the corresponding monomers (Fig. 5a and b). These shifts are ascribable to the energy hopping within the manifold of various emissive sites in the supramolecular fibers. More specifically, due to the inhomogeneous environment in the fibers, the emissive sites are continuously distributed at different energy levels, and the energy hopping among these sites results in the gradual red-shift of the fluorescence band with an increase in the delay time. Importantly, the red-shift of 1 (568 cm^−1^) was four times larger than that of 2 (132 cm^−1^) (Fig. 5c and d). The difference clearly reflects apparently distinct flexibility of supramolecular fibers due to the different degree of two-dimensional association. In other words, the flexible fibers of 1 involve a large conformational difference of constituent molecules, resulting in the lowering of the energy level of the excited chromophores (Fig. 5e and f). Such energy hopping along the flexible fiber was further verified by the fluorescence anisotropy measurements (Fig. S12†), which detect orientation change of the transition dipole moment of the excited chromophores accompanied with the energy hopping. Although the anisotropy decreased in both the fibers within the excited state lifetime, the fluorescence depolarization of 1 (0.28 → 0.16) was significantly larger than that of 2 (0.29 → 0.24). This result indicates that the excitation energy is relaxed to the chromophores disordered in the supramolecular structure of 1, which is the origin of the flexible fiber at the mesoscopic scale.

## Conclusions

Our previous studies have focused on the π–π-stacking driven supramolecular polymerization of barbiturates functionalized with relatively planar π-conjugated systems *via* supermacrocyclization based on hydrogen-bonding (“rosette pathway”). In this study, we have succeeded in developing barbiturate supramolecular polymers with high luminescence properties by direct supramolecular polymerization of barbiturates *via* a tape-like hydrogen bonded motif (“tape pathway”). The tape-like supramolecular polymerization was achieved by introducing twisted π-conjugated systems that prevent the competing rosette species from stacking. This design concept of monomers will be an important guideline for the creation of soft materials using barbiturates. The structural relaxation of the twisted π-conjugated systems in the excited state is suppressed through interchain aggregation of the tape-like supramolecular polymers, and accordingly AIE is observed. The modulation of the substitution position of the thiophene ring, the core of the twisted π-conjugated system, causes a slight difference in the polarity of the molecules, which results in a difference in the degree of interchain aggregation of the supramolecular polymers. As a result, we can obtain two soft materials, gel and nanosheet, and these materials exhibit different AIE properties depending on the monomer structures. The present study thus provides an important guideline for designing fluorescent functional materials and modulating their (photo)physical properties by conjugating twisted π-conjugated molecules and linearly hydrogen-bonded supramolecular scaffold.

## Data availability

All supporting data is provided in the ESI.†

## Author contributions

M. K. and H. S. performed the experiments. All authors contributed to interpret the results. S. Y. supervised the overall research. All authors contributed to the writing of the manuscript.

## Conflicts of interest

There are no conflicts to declare.

## Supplementary Material

SC-013-D1SC06246H-s001
