# Supplementary material for: Fluorescent supramolecular polymers of barbiturate dyes with thiophene-cored twisted π-systems
Source: Chem Sci. 2021 Dec 9;13(5):1281–7. doi: 10.1039/d1sc06246h (PMC8809409; doi:10.1039/d1sc06246h)
Supplement: SC-013-D1SC06246H-s001 [file SC-013-D1SC06246H-s001.pdf]

Electronic Supplementary Material (ESI) for Chemical Science.
This journal is © The Royal Society of Chemistry 2021

Supporting Information

Fluorescent Supramolecular Polymers of Barbiturate Dyes with Thiophene-Cored Twisted π-Systems

Maika Kawaura,[a] Takumi Aizawa,[a] Sho Takahashi,[a] Hiroshi Miyasaka,[b] Hikaru Sotome*[b] and Shiki Yagai*[c,d]

[a] Division of Advanced Science and Engineering, Graduate School of Science and Engineering, Chiba University, 1-33 Yayoi-cho, Inage-ku, Chiba 263-8522, Japan.

[b] Division of Frontier Materials Science, Graduate School of Engineering Science, Osaka University, 1-3 Machikaneyama, Toyonaka, Osaka 560-8531, Japan.

[c] Department of Applied Chemistry and Biotechnology, Graduate School of Engineering, Chiba University, 1-33 Yayoi-cho, Inage-ku, Chiba 263-8522, Japan.

[d] Institute for Global Prominent Research (IGPR), Chiba University, 1-33 Yayoi-cho, Inage-ku, Chiba 263-8522, Japan.

Corresponding authors:

Shiki Yagai; E-mail: yagai@faculty.chiba-u.jp

Hikaru Sotome: sotome@laser.chem.es.osaka-u.ac.jp

Table of Contents

1. General **S2**

2. Synthesis and Analytical Data **S4**

3. Supporting Table **S28**

4. Supporting Figures **S29**

5. Supporting References **S37**

1. General

Materials and Methods

Column chromatography was performed using 63–210 μm silica gel. All commercially available reagents and solvents were of reagent grade and used without purification. The solvents used to prepare supramolecular aggregates were all spectral grade and used without purification. ^{1}H and ^{13}C NMR spectra were recorded on Bruker-AVANCE III-400M spectrometer and chemical shifts are reported in ppm (δ) with the signal of tetramethylsilane (TMS) as the internal standard. APCI- and ESI-MS spectra were measured on an Exactive (Thermo Scientific). UV/vis spectra were recorded on a JASCO V760 spectrophotometer with Peltier device temperature-control unit. Fluorescence spectra were measured with JASCO FP-8300 with a JASCO ETC-815 temperature controller using a screw capped quartz cuvette with optical path length of 1.0 mm. FT-IR spectra were measured on JASCO FT/IR-4600 spectrometer. The emission quantum yields of solution sample of **1**, **2** and **3** were recorded on a Hamamatsu Quantaurus-QY spectrometer with an integrating sphere. Powder X-ray diffraction analysis was carried out on a Rigaku Rint-2200 X-ray diffractometer with monochromated CuKa radiation. All X-ray diffraction experiments were performed at room temperature. AFM images were acquired under ambient conditions using a Multimode 8 Nanoscope V microscope (Bruker Instruments) in peak force tapping (Scanasyst) mode. Silicon cantilevers (SCANASYST-AIR) with a spring constant of 0.4 N/m and frequency of 70 kHz (nominal value, Bruker, Japan) were used. Samples were prepared by spin-coating (3000 rpm, 1 min) solutions (10 μL) onto freshly cleaved HOPG. SEM images were acquired on JSM-6510 scanning electron microscopy. The samples were prepared by drop-casting aggregate solutions on a silicon substrate, dried under vacuum for 24 h, and sputtered with Pt using JFC-1600 (JEOL) Auto Fine Coater before observation.

Theoretical Calculations

All theoretical calculations were carried out with the Gaussian 16 software package.[S1] Geometry optimizations were performed using the density functional theory (DFT) with the CAM-B3LYP as functional and 6-31+G(d,p) basis set. Excitation energy was estimated using time-dependent DFT (TD-DFT) calculations at the CAM-B3LYP/6-31+G(d,p) levels of theory. The transition energy of the model compounds at fixed twist angles between aromatic units was also calculated using the same levels of theory.

Time-resolved fluorescence spectroscopy

Time-resolved fluorescence spectra (TRFS) and anisotropy (TRFA) were measured with a home-built setup based on a time-correlated single-photon counting (TCSPC) method. The pulsed light source was a Ti:sapphire laser (Spectra-Physics, Tsunami, 860 nm, 100 fs, 80 MHz). A small portion of the

output was picked up with a glass plate and detected as a starting pulse for TCSPC. The remaining major portion was converted into the second harmonics at 430 nm using a 2 mm beta barium borate crystal, and used for the excitation of the sample. The repetition rate was reduced to 8 MHz using an electro-optic modulator (Conoptics, Model 350). The fluorescence of the sample was detected by a photomultiplier tube (Hamamatsu, R3809U-50) after the spectral selection by a monochromator (Princeton Instruments, Acton SP-2150). The obtained signals were collected by a photon counting module (PicoQuant, PicoHarp 300). The sample solution was filled in a 1 cm quartz cell. The instrumental response function was determined by detecting a small portion of the excitation pulse scattered from colloidal solution, and evaluated as 45 ps. The reliability of the measurements was confirmed by measuring a fluorescence lifetime of the standard sample (Rhodamine B in water).[S2] In TRFS measurements, the polarization of the excitation pulse was set to the magic angle with respect to the fluorescence detection, and the spectral sensitivity of the detection system was corrected using fluorescent standard dyes.[S3] In TRFA measurements, the polarization of the excitation pulse was set to vertical to the optical table, and the parallel and perpendicular components ($I_{\parallel}, I_{\perp}$) of fluorescence were respectively extracted with a film polarizer in front of the monochromator. The time-dependent anisotropy was defined as $r(t) = \frac{I_{\parallel} - I_{\perp}}{I_{\parallel} + 2I_{\perp}}$ and calculated from the fluorescence signals.

2. Synthesis and Analytical Data

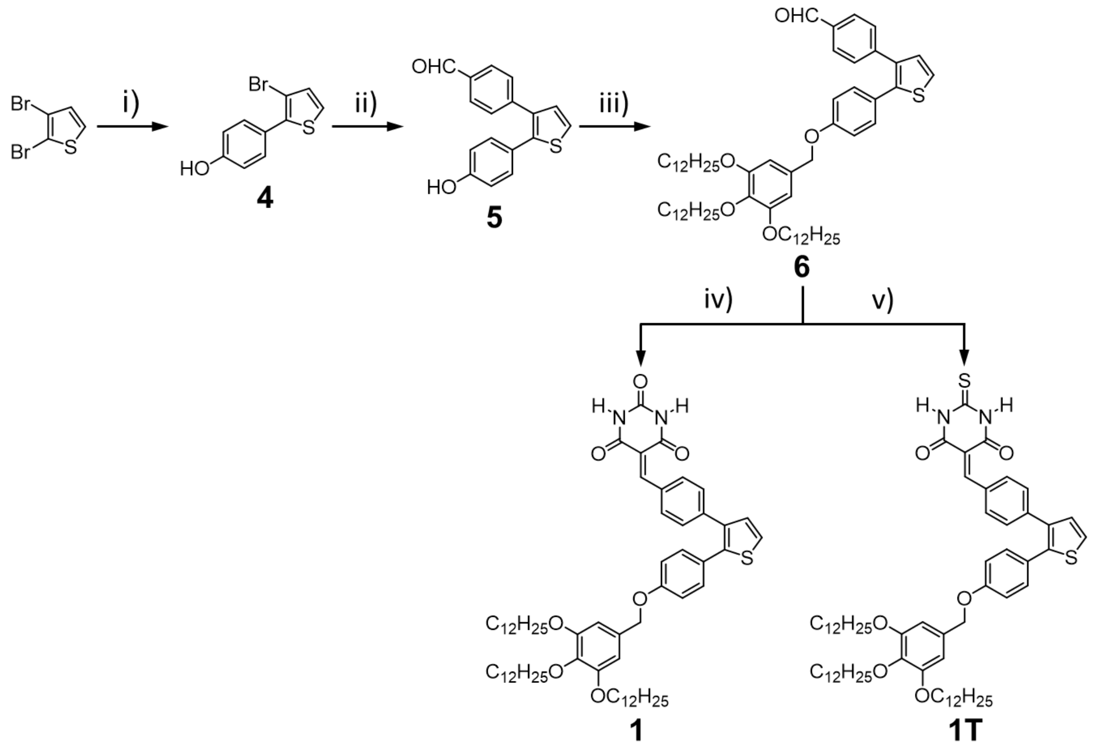

Scheme S1. Synthesis of **1** and **1T**. i) 4-hydroxyphenylboronic acid, $Pd(PPh_3)_4$, Na_2CO_3, 1,4-dioxane, 70 °C; ii) 4-formylphenylboronic acid, $Pd(PPh_3)_4$, K_2CO_3, 1,4-dioxane, 70 °C; iii) 3,4,5-tri(*n*-dodecyloxy)benzyl chloride[S4], K_2CO_3, DMF, 70 °C; iv) barbituric acid, EtOH, 70 °C, reflux; v) 2-thiobarbituric acid, EtOH, 70 °C, reflux.

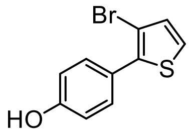

Synthesis of compound 4: 2,3-dibromothiophene (648 mg, 2.67 mmol), 4-hydroxyphenylboronic acid (368 mg, 2.67 mmol) and $Pd(PPh_3)_4$ (47.0 mg, 0.0406 mmol) were dissolved in dry 1,4-dioxane (20 mL). To this 1.09 M aq. Na_2CO_3 (10 mL, 10.9 mmol) was added and the mixture was stirred at 70 °C for 12 h under N_2 atmosphere. After the reaction mixture was diluted with AcOEt, the resulting solution was washed with water and brine, dried over Na_2SO_4, and evaporated. The residue was purified by column chromatography over silica gel (AcOEt:Hexane = 1:9) to give **4** as white solids (414 mg, 56% yield); ^{1}H NMR (400 MHz, $CDCl_3$, 293 K): δ = 7.54–7.52 (d, J = 8.7 Hz, 2H), 7.24–7.22 (d, J = 5.3 Hz, 1H), 7.03–7.02 (d, J = 5.4 Hz, 1H), 6.90–6.87 (d, J = 8.7 Hz, 2H), 4.84 (s, 1H). ^{13}C NMR (100 MHz, $CDCl_3$, 293 K): δ = 155.64, 138.03, 131.51, 130.62, 125.55, 124.39, 115.45, 107.11; HRMS (APCI): *m/z* calcd for $C_{10}H_8OBrS$ 254.9474 $[M+H]^+$, found 254.9477.

Synthesis of compound 5: Compound **4** (323 mg, 1.25 mmol), 4-formylphenylboronic acid (230 mg, 1.50 mmol) and $Pd(PPh_3)_4$ (22.0 mg, 0.0188 mmol) were dissolved in dry 1,4-dioxane (30 mL). To this 0.504 M aq. K_2CO_3 (10 mL, 5.04 mmol) was added and the mixture was stirred at 70 °C for 18 h under N_2 atmosphere. After the reaction mixture was diluted with AcOEt, the resulting solution was washed with water and brine, dried over Na_2SO_4, and evaporated. The resulting solid was purified by column chromatography over silica gel (AcOEt:Hexane = 1:2) to give **5** as yellow solids (233 mg, 65% yield). ^{1}H NMR (400 MHz, $CDCl_3$, 293 K): δ = 9.98 (s, 1H), 7.80–7.78 (d, J = 8.4 Hz, 2H), 7.45–7.43 (d, J = 8.3 Hz, 2H), 7.33–7.32 (d, J = 5.2 Hz, 1H), 7.18–7.14 (m, 3H), 6.78–6.74 (d, J = 8.7 Hz, 2H) , 4.83 (s, 1H); ^{13}C NMR (100 MHz, $CDCl_3$, 293 K): δ = 192.14, 155.61, 143.14, 140.51, 136.06, 134.56, 130.87, 130.43, 129.95, 129.78, 129.63, 128.07, 126.67, 126.25, 124.27, 115.92, 115.68; HRMS (APCI): *m/z* calcd for $C_{17}H_{13}O_2S$ 281.0631 $[M+H]^+$, found 281.0629.

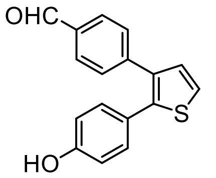

Synthesis of compound 6: Compound **5** (191 mg, 0.681 mmol), K_2CO_3 (404 mg, 2.92 mmol) and 5-(chloromethyl)-1,2,3-tris-(dodecyloxy)benzene[S4] (455 mg, 0.670 mmol) were dissolved in dry DMF (25 mL). The mixture was stirred at 70 °C for 3 h under N_2 atmosphere. After the reaction mixture was diluted with AcOEt:Hexane (1:4), the resulting solution was washed with water and brine, dried over Na_2SO_4, and evaporated. The resulting solid was purified by column chromatography over silica gel (AcOEt:Hexane = 1:9) to give **6** as yellow solids (598 mg, 97% yield). ^{1}H NMR (400 MHz, $CDCl_3$, 293 K): δ = 9.99 (s, 1H), 7.80–7.78 (d, J = 8.5 Hz, 2H), 7.46–7.44 (d, J = 8.2 Hz, 2H), 7.33–7.32 (d, J = 5.2 Hz, 1H), 7.22–7.20 (d, J = 8.8 Hz, 2H), 7.19–7.17 (d, J = 5.3 Hz, 1H), 6.91–6.88 (d, J = 8.9 Hz, 2H), 6.61 (s, 2H), 4.93 (s, 2H), 3.99–3.93 (m, 6H), 1.82–1.71 (m, 6H), 1.55–1.42 (m, 6H), 1.36–1.26 (m, 48H), 0.89–0.86 (m, 9H); ^{13}C NMR (100 MHz, $CDCl_3$, 293 K): δ = 191.85, 158.67, 153.35, 143.05, 140.49, 138.10, 136.09, 134.66, 131.51, 130.64, 129.86, 129.62, 126.42, 124.29, 115.03, 106.27, 73.46, 70.51, 69.17, 31.96, 31.95, 30.37, 29.78, 29.76, 29.72, 29.67, 29.44, 29.43, 29.39, 26.16, 26.13, 22.71, 14.14; HRMS (APCI): *m/z* calcd for $C_{60}H_{91}O_5S$ 923.6582 $[M+H]^+$, found 923.6579.

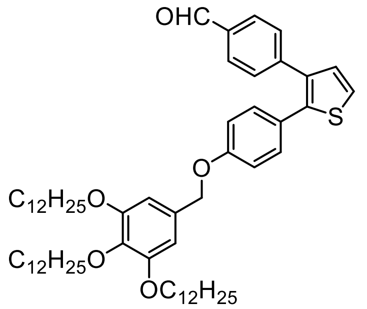

Synthesis of compound 1: Compound **6** (218 mg, 0.236 mmol) and barbituric acid (147 mg, 1.15 mmol) in EtOH (10 mL) were stirred at 70 °C for 18 h under reflux. The reaction mixture was cooled to room temperature and the resulting precipitates were collected by filtration and washed with hot ethanol repeatedly. The residue was dissolved in chloroform, and ethanol was added at 0 °C. The resulting precipitates were collected by centrifugation to give pure compound **1** as yellow solids (185 mg, 76% yield). ^{1}H NMR (400 MHz, $CDCl_3$, 293 K): δ= 8.54 (s, 1H), 8.31 (s, 1H), 8.23 (s, 1H), 8.18–8.15 (d, J = 8.5 Hz, 2H), 7.42–7.40 (d, J = 8.5 Hz, 2H), 7.34–7.32 (d, J = 5.3 Hz, 1H), 7.25–7.23 (d, J = 8.8 Hz, 2H), 7.22–7.20 (d, J = 5.3 Hz, 1H), 6.92–6.90 (d, J = 8.8 Hz, 2H), 6.61 (s, 2H), 4.94 (s, 2H), 3.99–3.93 (m, 6H), 1.82–1.73 (m, 6H), 1.48–1.42 (m, 6H), 1.28–1.23 (m, 48H), 0.89–0.86 (m, 9H); ^{13}C NMR (100 MHz, $CDCl_3$, 293 K): δ= 163.11, 160.63, 160.12, 158.74, 153.34, 148.85, 142.99, 141.13, 137.96, 135.96, 135.48, 131.53, 130.69, 130.34, 129.75, 129.01, 126.38, 124.41, 115.25, 115.06, 106.13, 73.51, 70.46, 69.12, 31.95, 30.34, 29.79, 29.77, 29.73, 29.68, 29.45, 29.42, 29.39, 26.16, 26.13, 22.72, 14.15; HRMS (ESI): *m/z* calcd for $C_{64}H_{92}O_7N_2ClS$ 1067.6308 $[M+Cl]^-$, found 1067.6337.

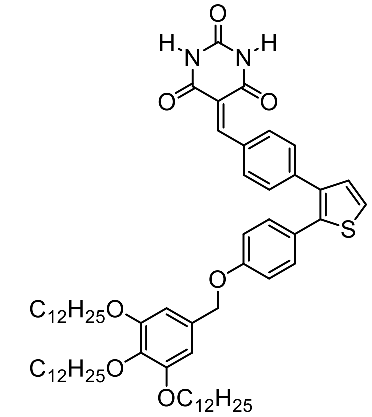

Synthesis of compound 1T: Compound **6** (36 mg, 0.039 mmol) and 2-thiobarbituric acid (6.1 mg, 0.042 mmol) in EtOH (2 mL) were stirred at 70 °C for 16 h under reflux. The reaction mixture was cooled to room temperature and the resulting precipitates were collected by filtration and washed with hot methanol repeatedly to give pure compound **1T** as orange solids (34 mg, 83% yield). ^{1}H NMR (400 MHz, $CDCl_3$, 293 K): δ= 9.28 (s, 1H), 9.24 (s, 1H), 8.55 (s, 1H), 8.23–8.21 (d, J = 8.5 Hz, 2H), 7.43–7.41 (d, J = 8.5 Hz, 2H), 7.34–7.32 (d, J = 5.2 Hz, 1H), 7.25–7.22 (d, J = 8.8 Hz, 2H), 7.22–7.21 (d, J = 5.3 Hz, 1H), 6.92–6.90 (d, J = 8.9 Hz, 2H), 6.61 (s, 2H), 4.95 (s, 2H), 3.99–3.94 (m, 6H), 1.82–1.73 (m, 6H), 1.49–1.42 (m, 6H), 1.35–1.26 (m, 48H), 0.89–0.86 (m, 9H); ^{13}C NMR (100 MHz, $CDCl_3$, 293 K): δ= 175.81, 161.36, 160.67, 158.77, 158.72, 153.34, 143.54, 141.41, 137.93, 135.95, 135.90, 131.51, 130.71, 130.42, 129.70, 129.12, 126.32, 124.51, 115.13, 115.09, 106.11, 73.52, 70.45, 69.12, 31.98, 31.96, 30.34, 29.80, 29.74, 29.69, 29.46, 29.40, 26.14, 22.73, 14.17; HRMS (APCI): *m/z* calcd for $C_{64}H_{93}O_6N_2S_2$ 1049.6470 $[M+H]^+$, found 1049.6472.

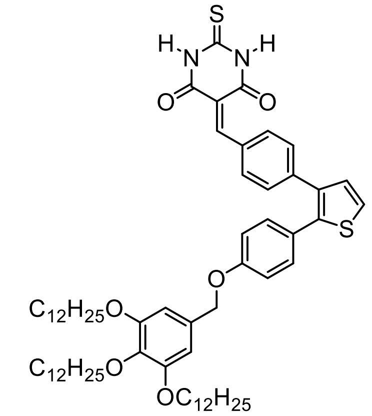

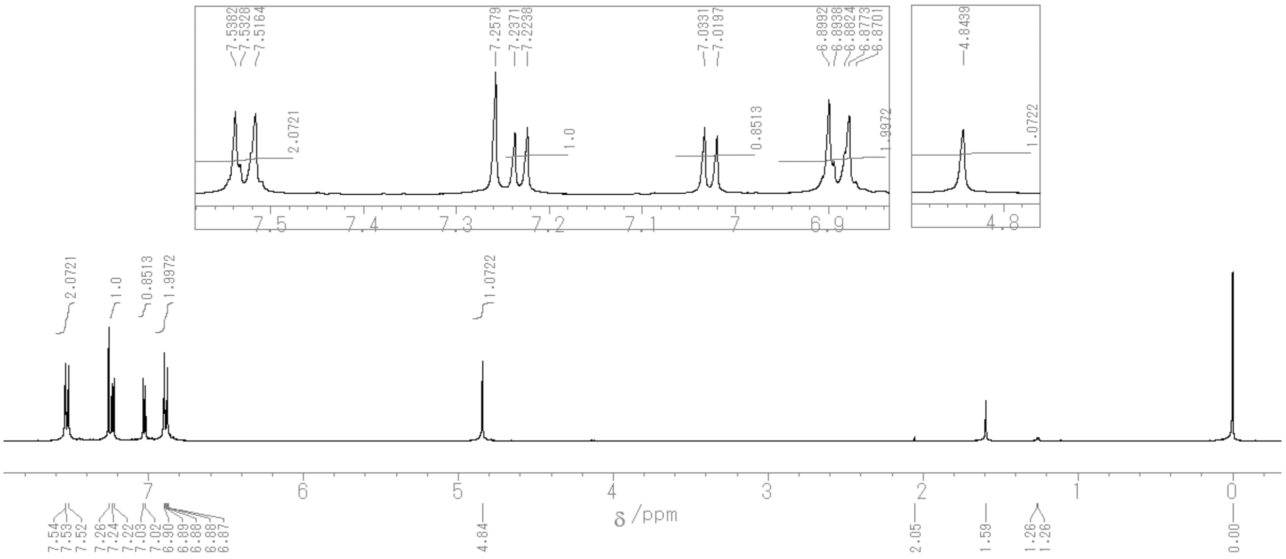

Chart S1. ^{1}H NMR spectrum of compound **4** in $CDCl_3$ at 293 K.

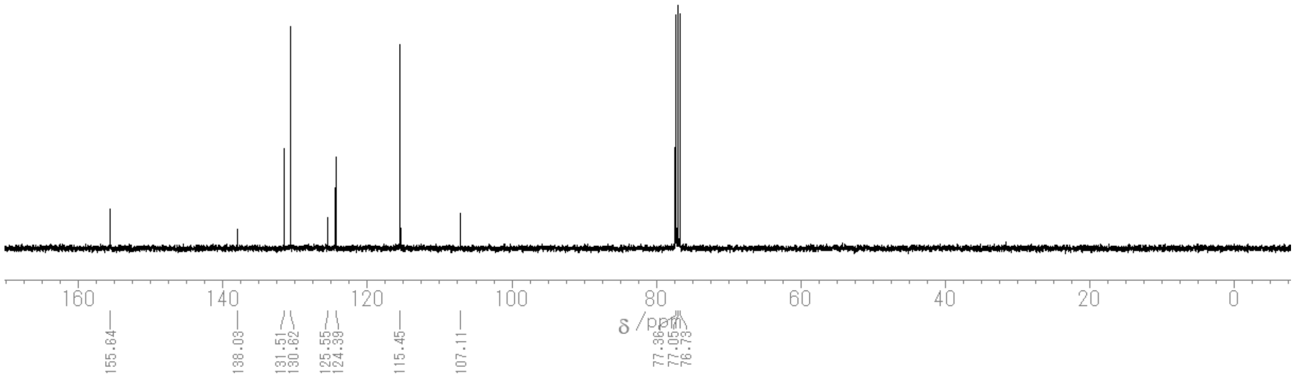

Chart S2. ^{13}C NMR spectrum of compound **4** in $CDCl_3$ at 293 K.

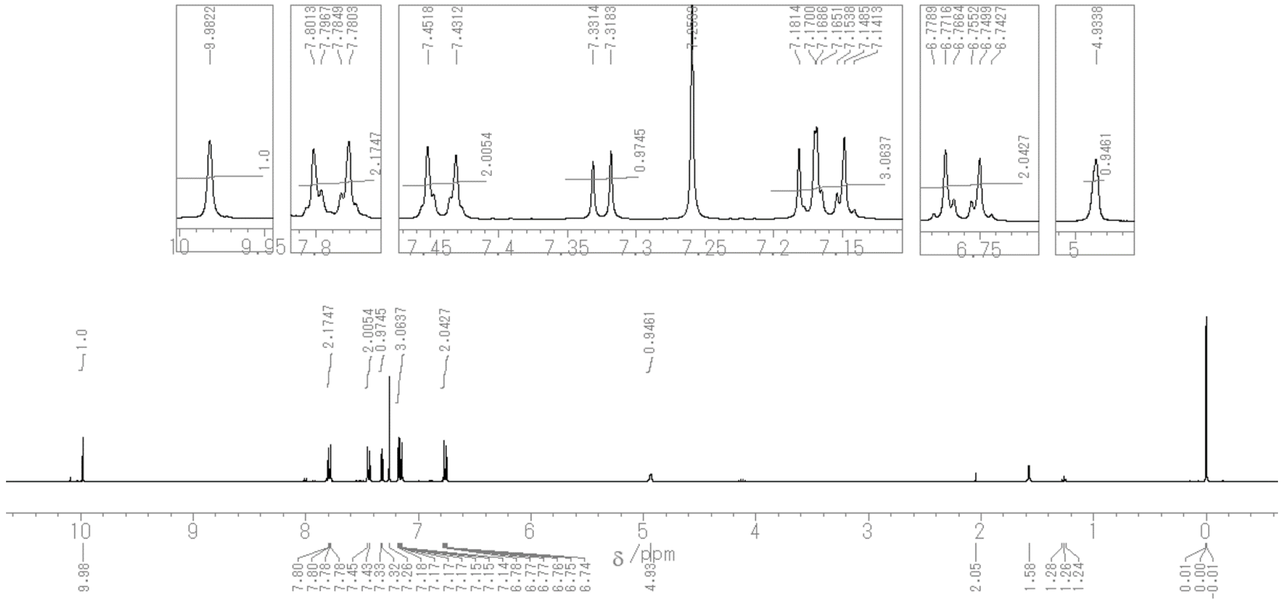

Chart S3. ^{1}H NMR spectrum of compound **5** in $CDCl_3$ at 293 K.

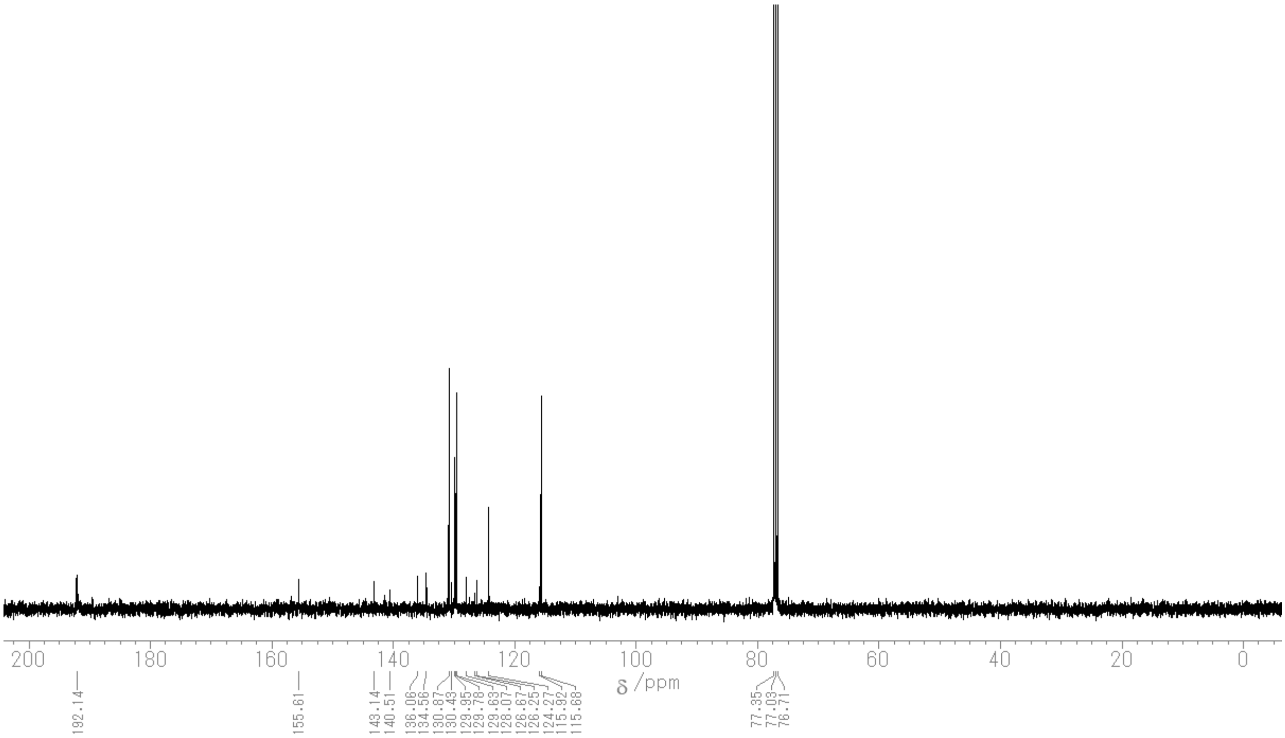

Chart S4. ^{13}C NMR spectrum of compound **5** in $CDCl_3$ at 293 K.

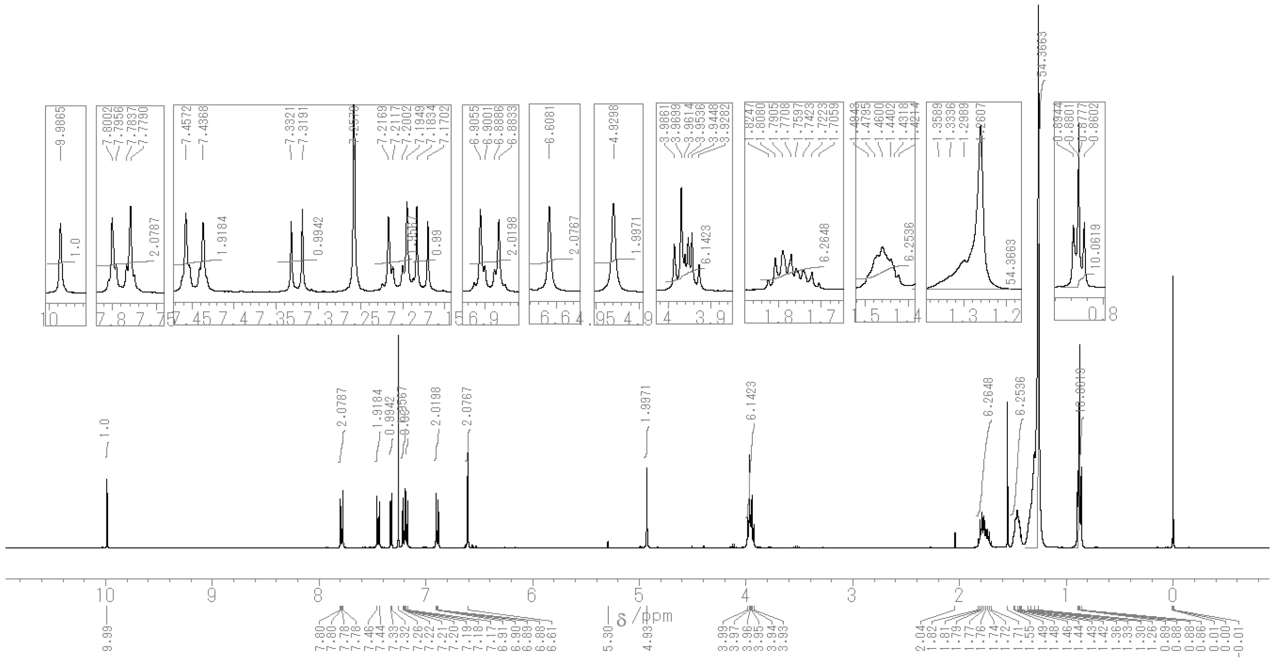

Chart S5. ^{1}H NMR spectrum of compound **6** in $CDCl_3$ at 293 K.

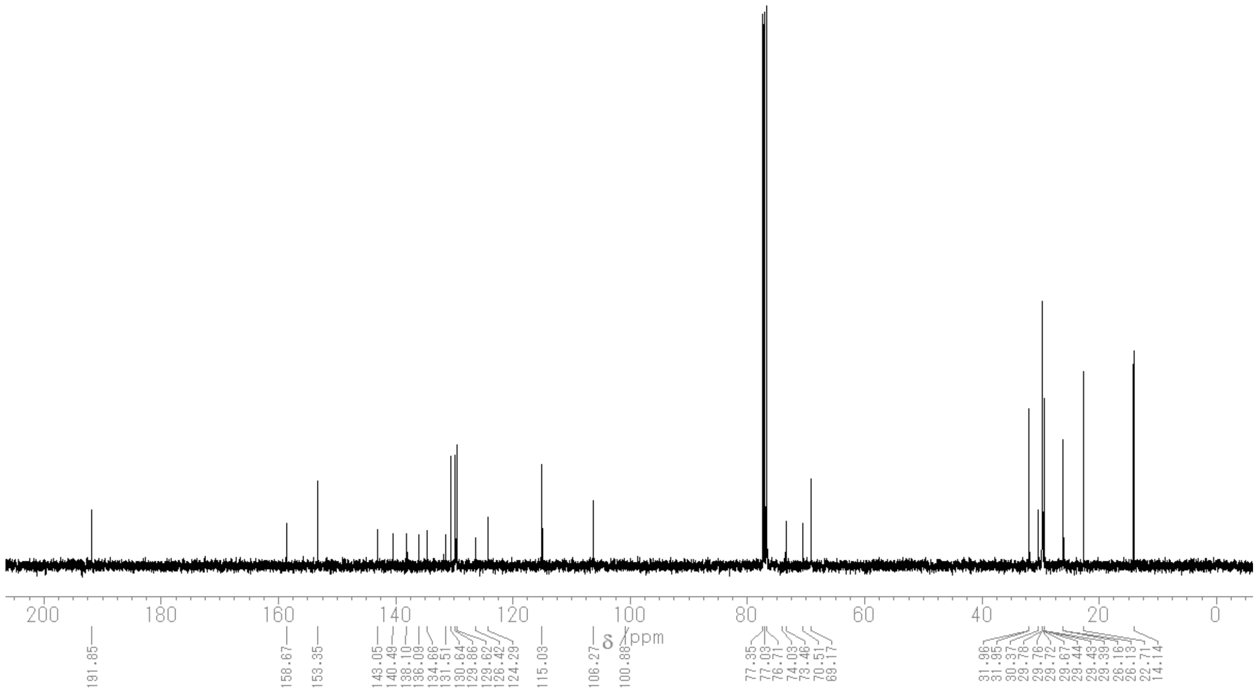

Chart S6. ^{13}C NMR spectrum of compound **6** in $CDCl_3$ at 293 K.

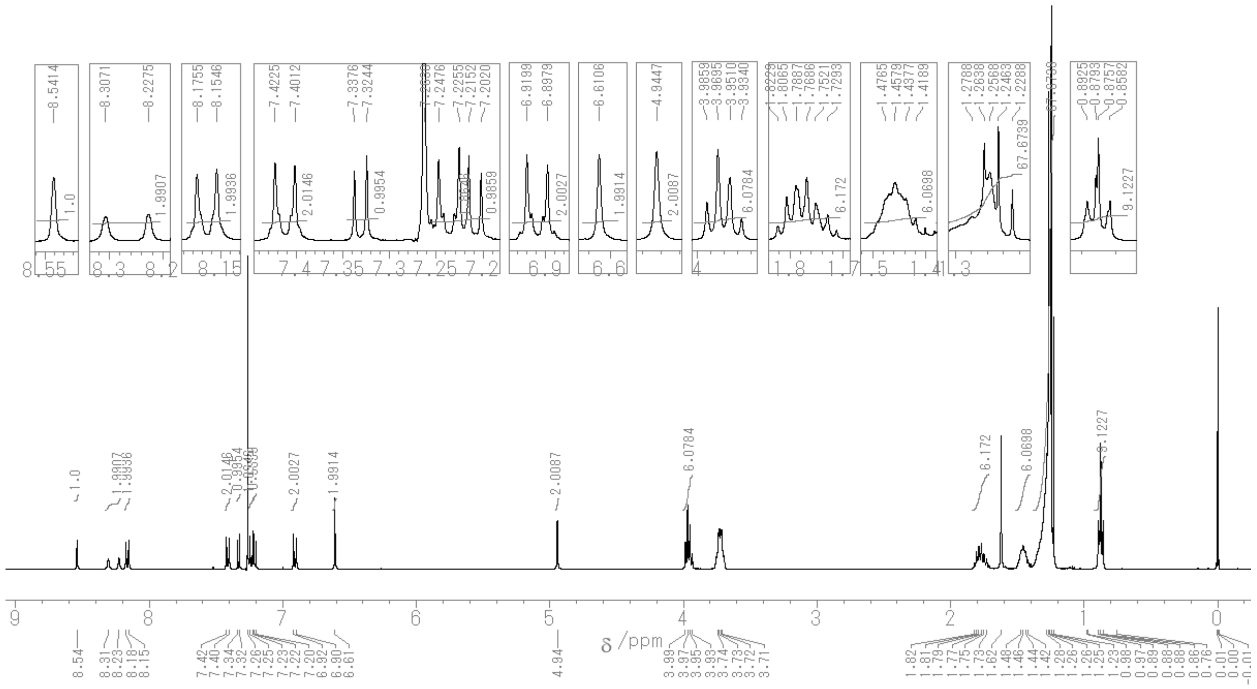

Chart S7. ^{1}H NMR spectrum of compound **1** in $CDCl_3$ at 293 K.

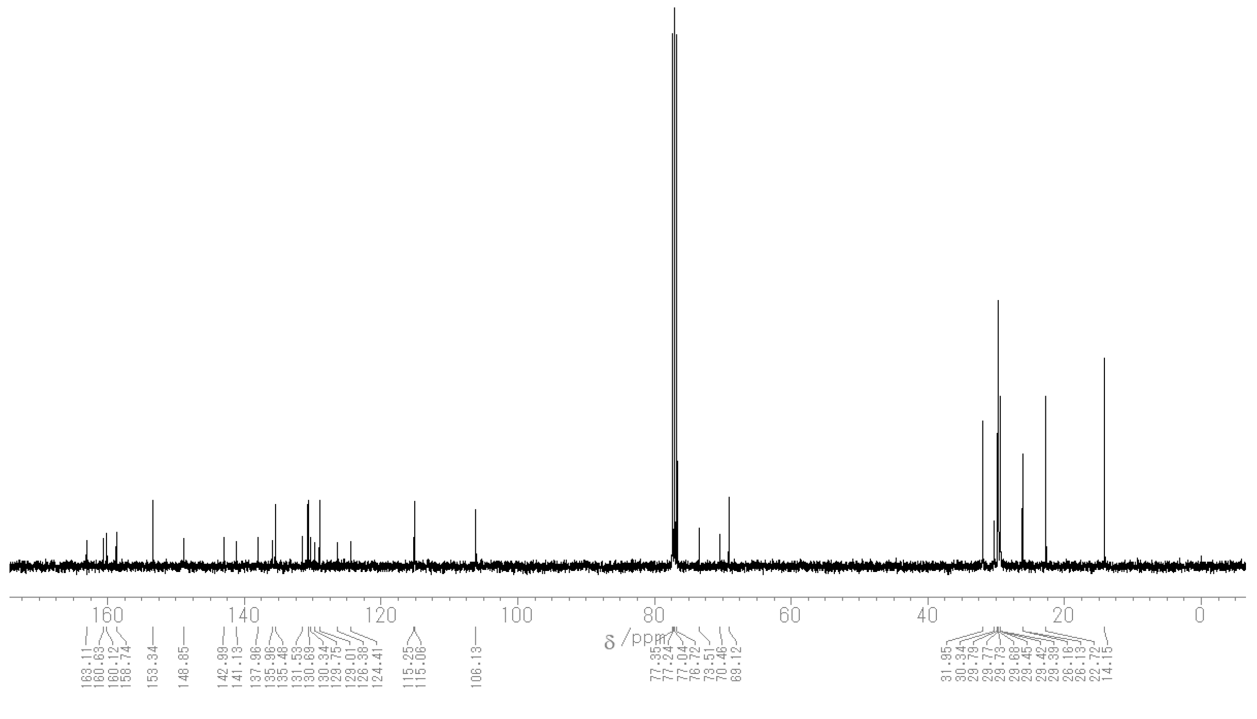

Chart S8. ^{13}C NMR spectrum of compound **1** in $CDCl_3$ at 293 K.

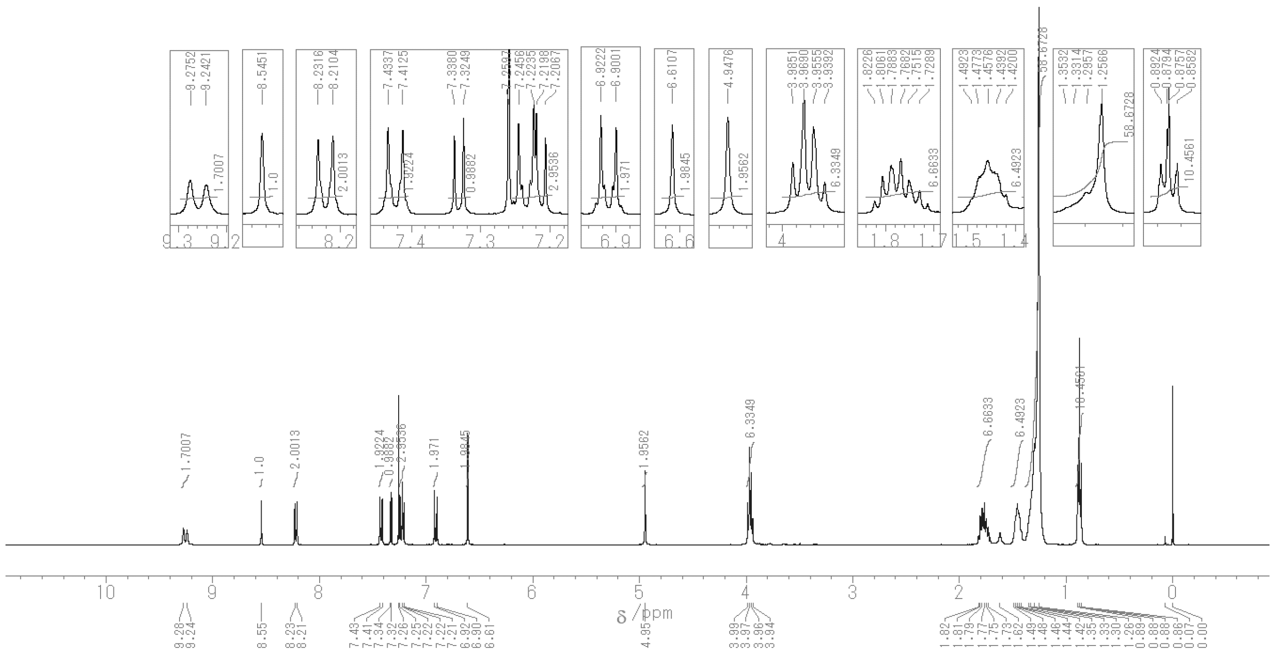

Chart S9. ^{1}H NMR spectrum of compound **1T** in $CDCl_3$ at 293 K.

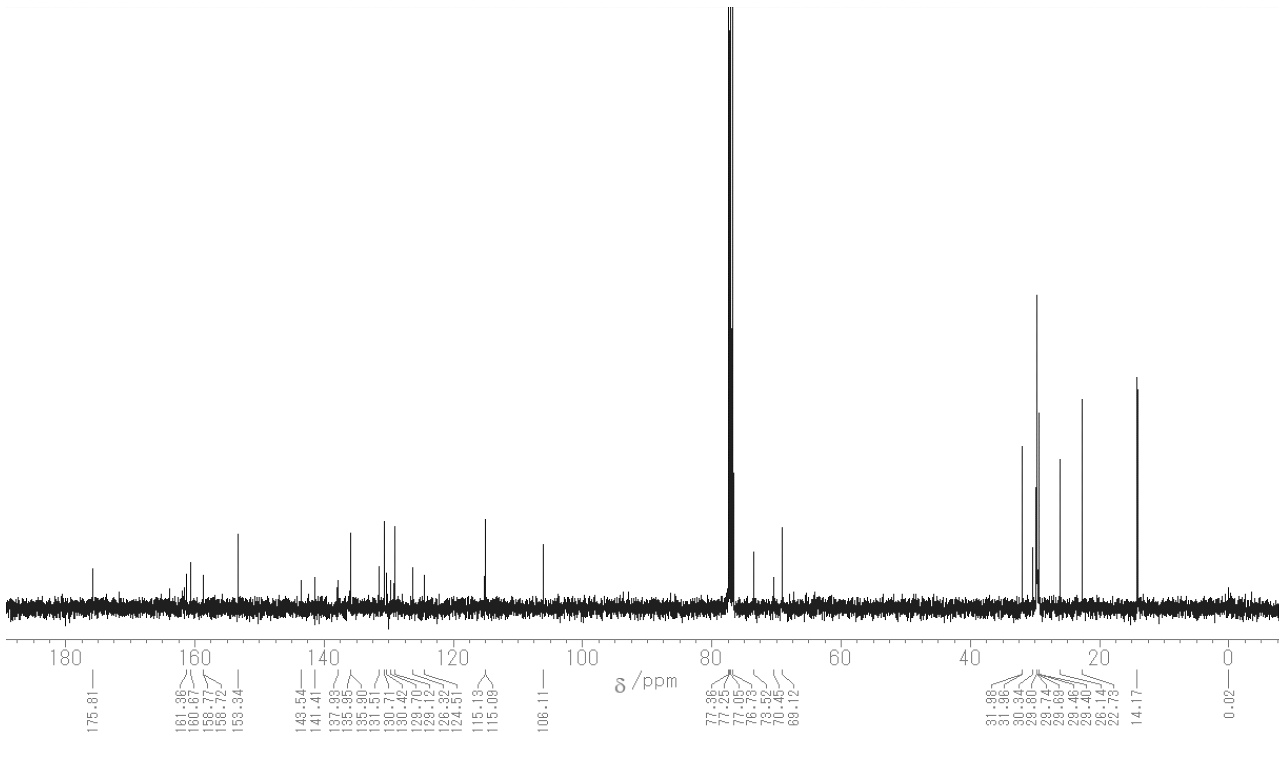

Chart S10. ^{13}C NMR spectrum of compound **1T** in $CDCl_3$ at 293 K.

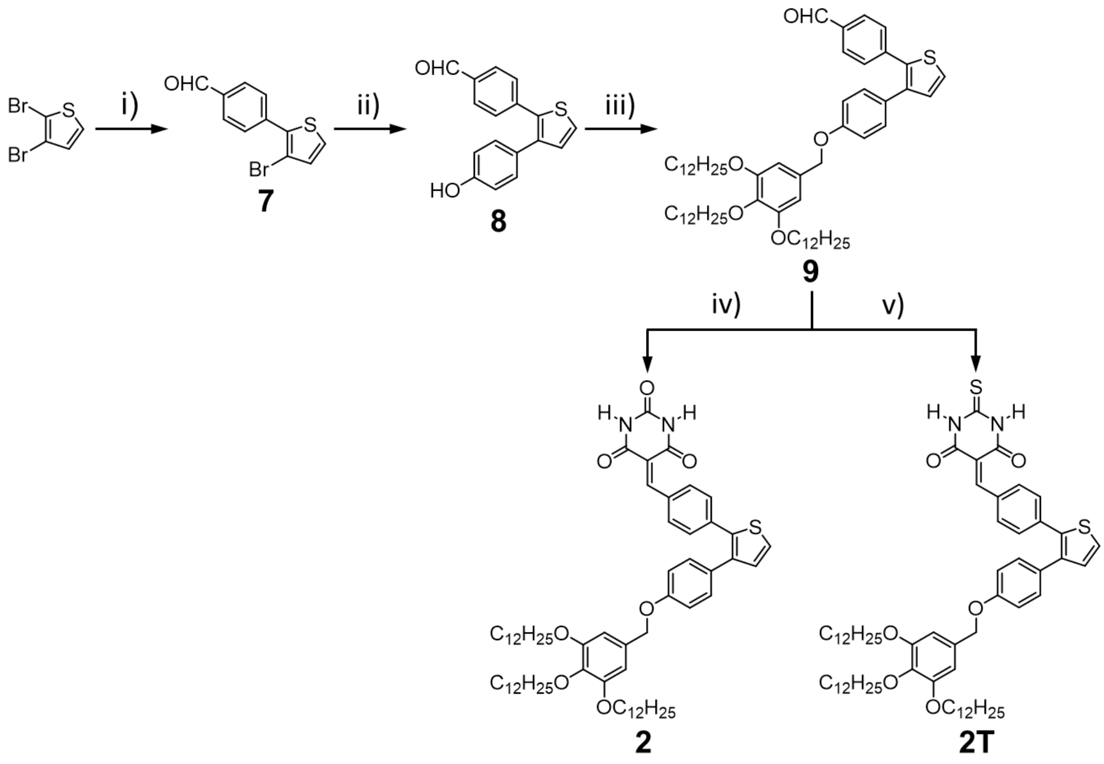

Scheme S2. Synthesis of **2** and **2T**. i) 4-formylphenylboronic acid, $Pd(PPh_3)_4$, Na_2CO_3, 1,4-dioxane, 70 °C; ii) 4-hydroxylphenylboronic acid, $Pd(PPh_3)_4$, K_2CO_3, 1,4-dioxane, 70 °C; iii) 3,4,5-tri(*n*-dodecyloxy)benzyl chloride[S4], K_2CO_3, DMF, 70 °C; iv) barbituric acid, EtOH, 70 °C, reflux; v) 2-thiobarbituric acid, EtOH, 70 °C, reflux.

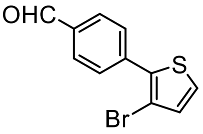

Synthesis of compound 7: 2,3-dibromothiophene (663 mg, 2.68 mmol), 4-formylphenylboronic acid (401 mg, 2.68 mmol) and $Pd(PPh_3)_4$ (44.0 mg, 0.0380 mmol) were dissolved in dry 1,4-dioxane (20 mL). To this 1.08 M aq. Na_2CO_3 (10 mL, 10.8 mmol) was added and the mixture was stirred at 70 °C for 13 h under N_2 atmosphere. After the reaction mixture was diluted with AcOEt, the resulting solution was washed with water and brine, dried over Na_2SO_4, and evaporated. The residue was purified by column chromatography over silica gel (AcOEt:Hexane = 1:5) to give **7** as white solids (451 mg, 63% yield). ^{1}H NMR (400 MHz, $CDCl_3$, 293 K): δ= 10.06 (s, 1H), 7.96–7.93 (d, J = 8.2 Hz, 2H), 7.87–7.85 (d, J = 8.4 Hz, 2H), 7.38–7.37 (d, J = 5.5 Hz, 1H), 7.11–7.10 (d, J = 5.5 Hz, 1H); ^{13}C NMR (100 MHz, $CDCl_3$, 293 K): δ= 191.60, 138.85, 136.66, 135.60, 132.28, 129.88, 129.41, 126.40, 108.92, ; HRMS (APCI): *m/z* calcd for $C_{11}H_8OBrS$ 266.9429 $[M+H]^+$, found 266.9475.

Synthesis of compound 8: Compound **7** (308 mg, 1.15 mmol), 4-hydroxyphenylboronic acid (210 mg, 1.52 mmol) and $Pd(PPh_3)_4$ (33.0 mg, 0.0285 mmol) were dissolved in dry 1,4-dioxane (20 mL). To this 0.461 M aq. K_2CO_3 (10 mL, 4.61 mmol) was added and the mixture was stirred at 70 °C for 17 h under N_2 atmosphere. After the reaction mixture was diluted with AcOEt, the resulting solution was washed with water and brine, dried over Na_2SO_4, and evaporated. The resulting solid was purified by column chromatography over silica gel (AcOEt:Hexane = 1:2) to give **8** as yellow solids (290 mg, 90% yield). ^{1}H NMR (400 MHz, $CDCl_3$, 293 K): δ = 9.97 (s, 1H), 7.78–7.76 (d, J = 8.5 Hz, 2H), 7.47–7.45 (d, J = 8.2 Hz, 2H), 7.40–7.39 (d, J = 5.2 Hz, 1H), 7.16–7.13 (d, J = 8.7 Hz, 2H), 7.14–7.13 (d, J = 5.1 Hz, 1H), 6.80–6.77 (d, J = 8.7 Hz, 2H), 5.03 (s, 1H); ^{13}C NMR (100 MHz, $CDCl_3$, 293 K): δ = 192.12, 155.18, 141.09, 139.61, 136.11, 134.72, 133.82, 131.05, 130.46, 130.01, 129.57, 128.59, 125.65, 115.62; HRMS (APCI): *m/z* calcd for $C_{17}H_{13}O_2S$ 281.0631 $[M+H]^+$, found 281.0629.

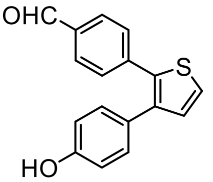

Synthesis of compound 9: Compound **8** (149 mg, 0.531 mmol), K_2CO_3 (310 mg, 2.24 mmol) and 5-(chloromethyl)-1,2,3-tris-(dodecyloxy)benzene[S4] (360 mg, 0.530 mmol) were dissolved in dry DMF (15 mL). The mixture was stirred at 70 °C for 15 h under N_2 atmosphere. After the reaction mixture was diluted with AcOEt:Hexane (1:4), the resulting solution was washed with water and brine, dried over Na_2SO_4, and evaporated. The resulting solid was purified by column chromatography over silica gel (AcOEt:Hexane = 1:9) to give **9** as pale yellow solids (365 mg, 75% yield). ^{1}H NMR (400 MHz, $CDCl_3$, 293 K): δ = 9.98 (s, 1H), 7.78–7.76 (d, J = 8.5 Hz, 2H), 7.48–7.46 (d, J = 8.2 Hz, 2H), 7.41–7.40 (d, J = 5.2 Hz, 1H), 7.21–7.19 (d, J = 8.8 Hz, 2H), 7.16–7.14 (d, J = 5.2 Hz, 1H), 6.93–6.91 (d, J = 8.9 Hz, 2H), 6.62 (s, 2H), 4.94 (s, 2H), 3.99–3.93 (m, 6H), 1.83–1.71 (m, 6H), 1.46–1.42 (m, 6H), 1.30–1.26 (m, 48H), 0.89–0.86 (m, 9H); ^{13}C NMR (100 MHz, $CDCl_3$, 293 K): δ = 191.64, 158.24, 153.34, 140.90, 139.51, 138.08, 136.19, 134.88, 131.64, 131.01, 130.26, 129.89, 129.56, 128.79, 125.60, 114.97, 106.30, 73.46, 70.50, 69.18, 31.95, 30.37, 29.78, 29.73, 29.68, 29.45, 29.39, 26.13, 22.71, 14.14; HRMS (APCI): *m/z* calcd for $C_{60}H_{91}O_5S$ 923.6582 $[M+H]^+$, found 923.6577.

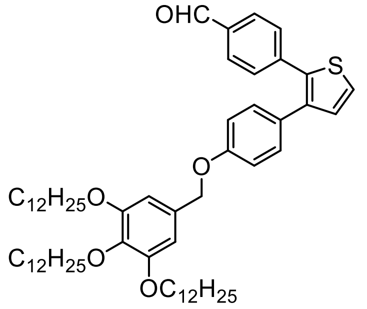

Synthesis of compound 2: Compound **9** (205 mg, 0.222 mmol) and barbituric acid (154 mg, 1.20 mmol) in EtOH (10 mL) were stirred at 70 °C for 18 h under reflux. The reaction mixture was cooled to room temperature and the resulting precipitates were collected by filtration and washed with hot ethanol repeatedly. The residue was dissolved in chloroform, and ethanol was added at 0 °C. The resulting precipitates were collected by centrifugation to give pure compound **2** as orange-yellow solids (183 mg, 81% yield). ^{1}H NMR (400 MHz, $CDCl_3$, 293 K): δ = 8.52 (s, 1H), 8.14–8.12 (d, J = 8.5 Hz, 2H), 8.07 (s, 1H), 8.04 (s, 1H), 7.43–7.41 (d, J = 8.6 Hz, 2H), 7.42–7.41 (d, J = 5.2 Hz, 1H), 7.24–7.21 (d, J = 8.7 Hz, 2H), 7.15–7.14 (d, J = 5.3 Hz, 1H), 6.95–6.92 (d, J = 8.8 Hz, 2H), 6.62 (s, 2H), 4.96 (s, 2H), 3.99–3.94 (m, 6H), 1.82–1.73 (m, 6H), 1.49–1.42 (m, 6H), 1.35–1.26 (m, 48H), 0.89–0.86 (m, 9H); ^{13}C NMR (100 MHz, $CDCl_3$, 293 K): δ = 163.08, 160.59, 159.78, 158.30, 153.33, 148.84, 140.88, 140.09, 137.89, 136.32, 135.47, 134.81, 131.66, 131.30, 130.62, 130.30, 128.79, 128.73, 126.08, 115.35, 114.99, 106.12, 73.52, 70.43, 69.10, 31.98, 31.95, 30.34, 29.80, 29.78, 29.74, 29.69, 29.46, 29.42, 29.40, 26.16, 26.14, 22.73, 14.17; HRMS (ESI): *m/z* calcd for $C_{64}H_{92}O_7N_2ClS$ 1067.6308 $[M+Cl]^-$, found 1067.7157.

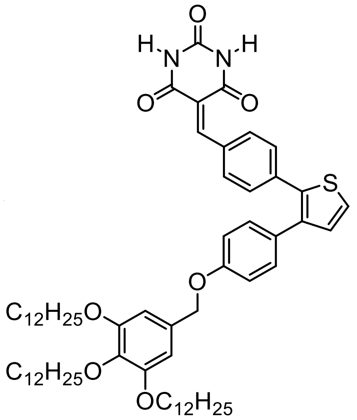

Synthesis of compound 2T: Compound **9** (50 mg, 0.054 mmol) and 2-thiobarbituric acid (7.7 mg, 0.053 mmol) in EtOH (3 mL) were stirred at 70 °C for 6 h under reflux. The reaction mixture was cooled to room temperature and the resulting precipitates were collected by filtration and washed with hot methanol repeatedly to give pure compound **2T** as orange solids (30 mg, 53% yield). ^{1}H NMR (400 MHz, $CDCl_3$, 293 K): δ = 9.06 (s, 1H), 8.99 (s, 1H), 8.51 (s, 1H), 8.19–8.16 (d, J = 8.3 Hz, 2H), 7.44–7.42 (m, 3H), 7.24–7.21 (d, J = 8.8 Hz, 2H), 7.15–7.14 (d, J = 5.2 Hz, 1H), 6.95–6.93 (d, J = 8.9 Hz, 2H), 6.62 (s, 2H), 4.96 (s, 2H), 3.99–3.94 (m, 6H), 1.83–1.73 (m, 6H), 1.49–1.42 (m, 6H), 1.35–1.26 (m, 48H), 0.89–0.86 (m, 9H); ^{13}C NMR (100 MHz, $CDCl_3$, 293 K): δ = 158.26, 153.34, 139.69, 137.97, 136.93, 134.34, 133.19, 131.93, 131.76, 131.00, 129.70, 129.61, 129.51, 129.11, 127.81, 126.58, 119.61, 116.84, 112.13, 106.19, 73.47, 70.49, 69.15, 31.98, 31.96, 30.38, 29.80, 29.78, 29.74, 29.70, 29.47, 29.44, 29.41, 26.18, 26.15, 22.73, 20.85, 14.16; HRMS (APCI): *m/z* calcd for $C_{64}H_{93}O_6N_2S_2$ 1049.6470 $[M+H]^+$, found 1049.6473.

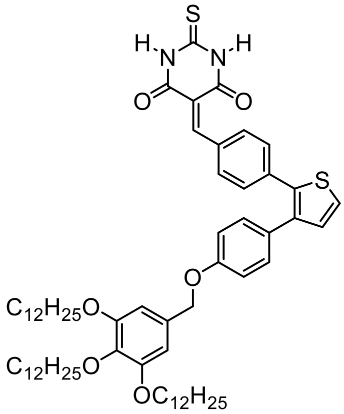

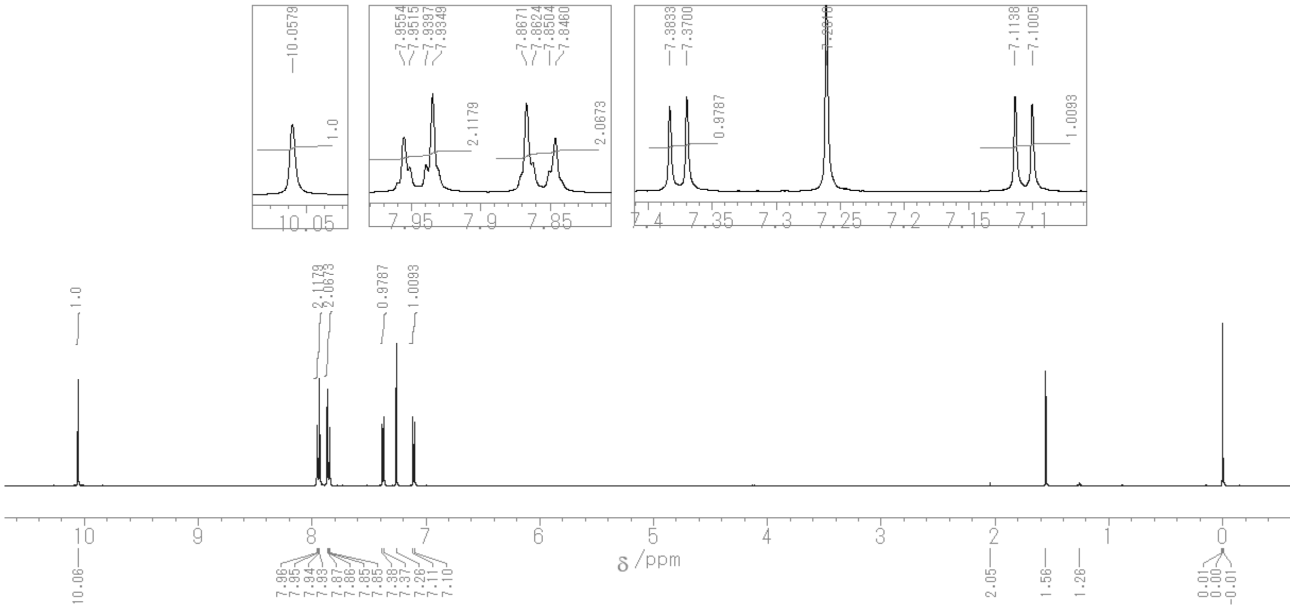

Chart S11. ^{1}H NMR spectrum of compound **7** in $CDCl_3$ at 293 K.

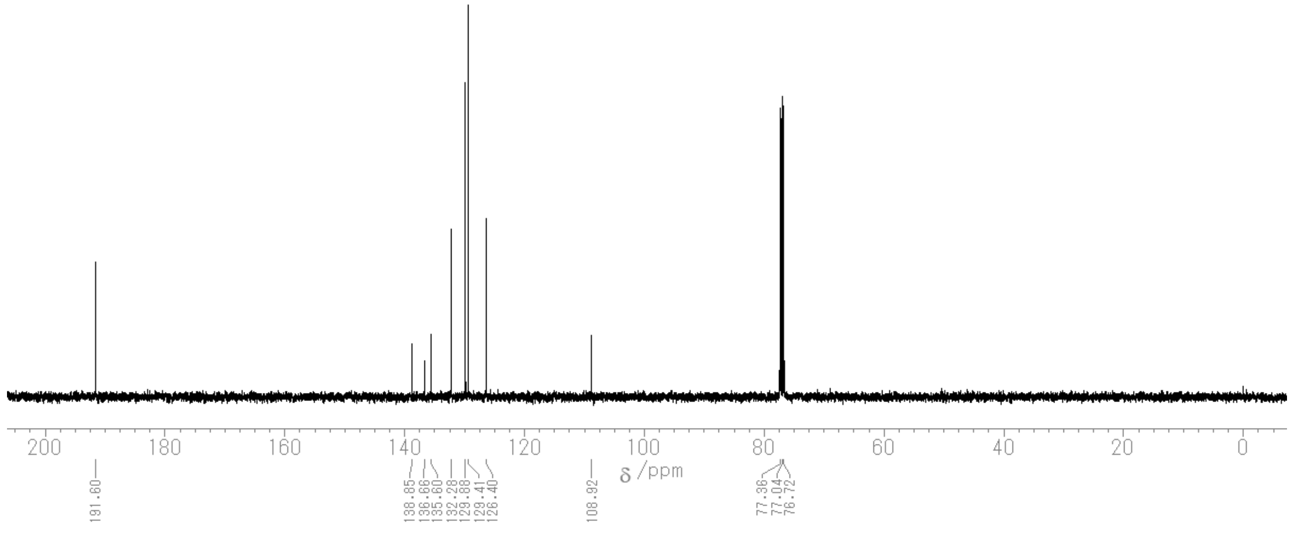

Chart S12. ^{13}C NMR spectrum of compound **7** in $CDCl_3$ at 293 K.

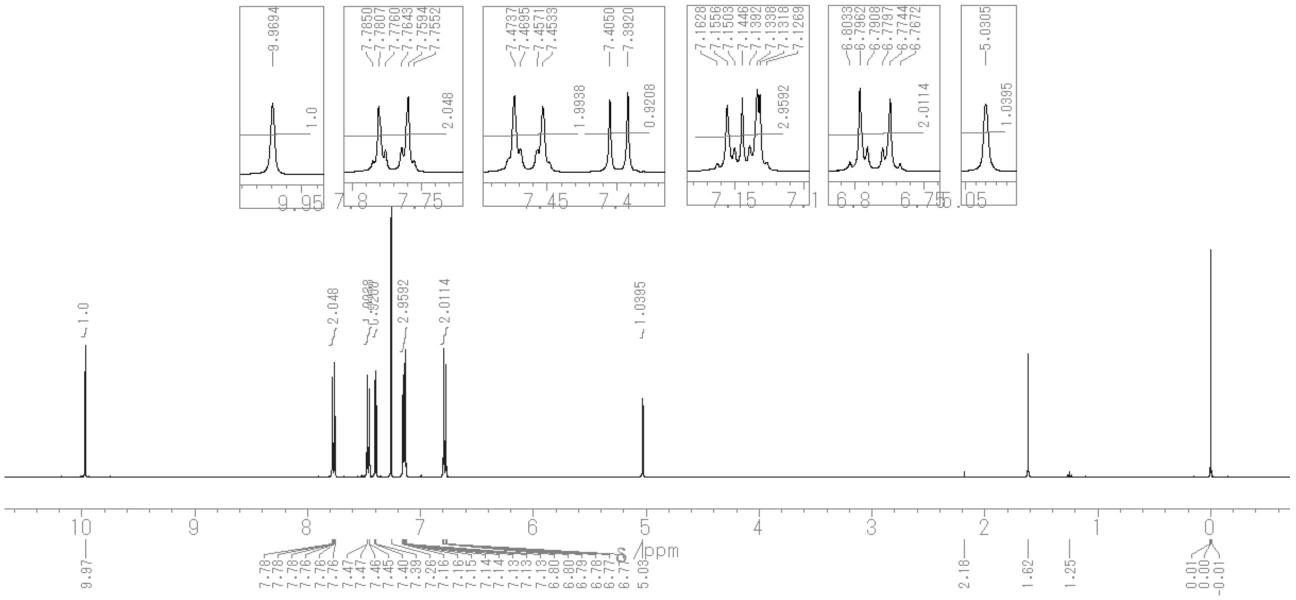

Chart S13. ^{1}H NMR spectrum of compound **8** in $CDCl_3$ at 293 K.

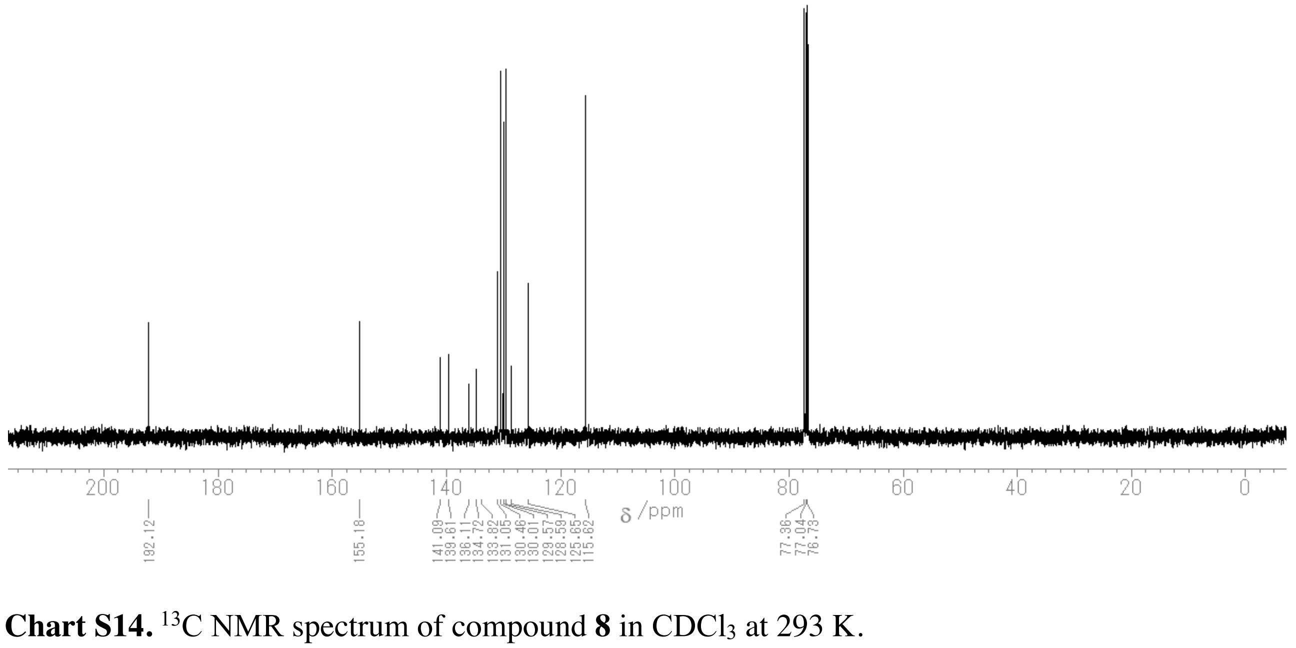

Chart S14. ^{13}C NMR spectrum of compound **8** in $CDCl_3$ at 293 K.

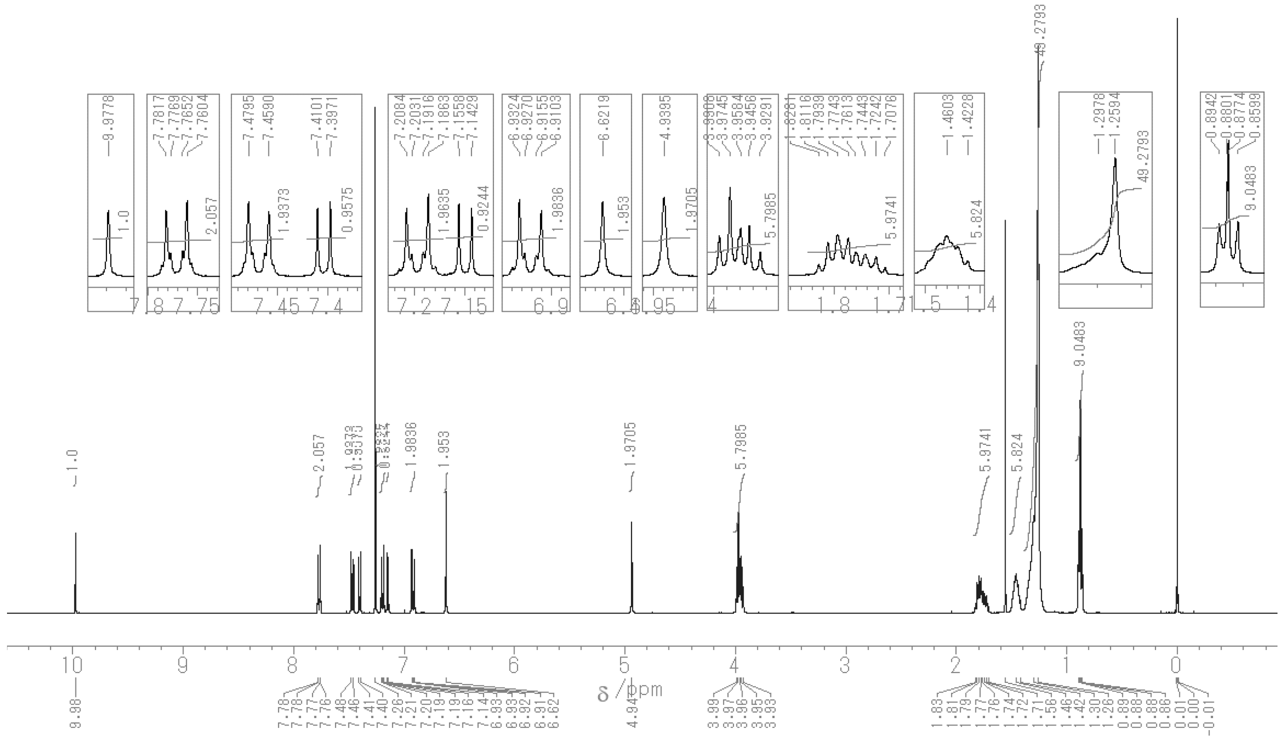

Chart S15. ^{1}H NMR spectrum of compound **9** in $CDCl_3$ at 293 K.

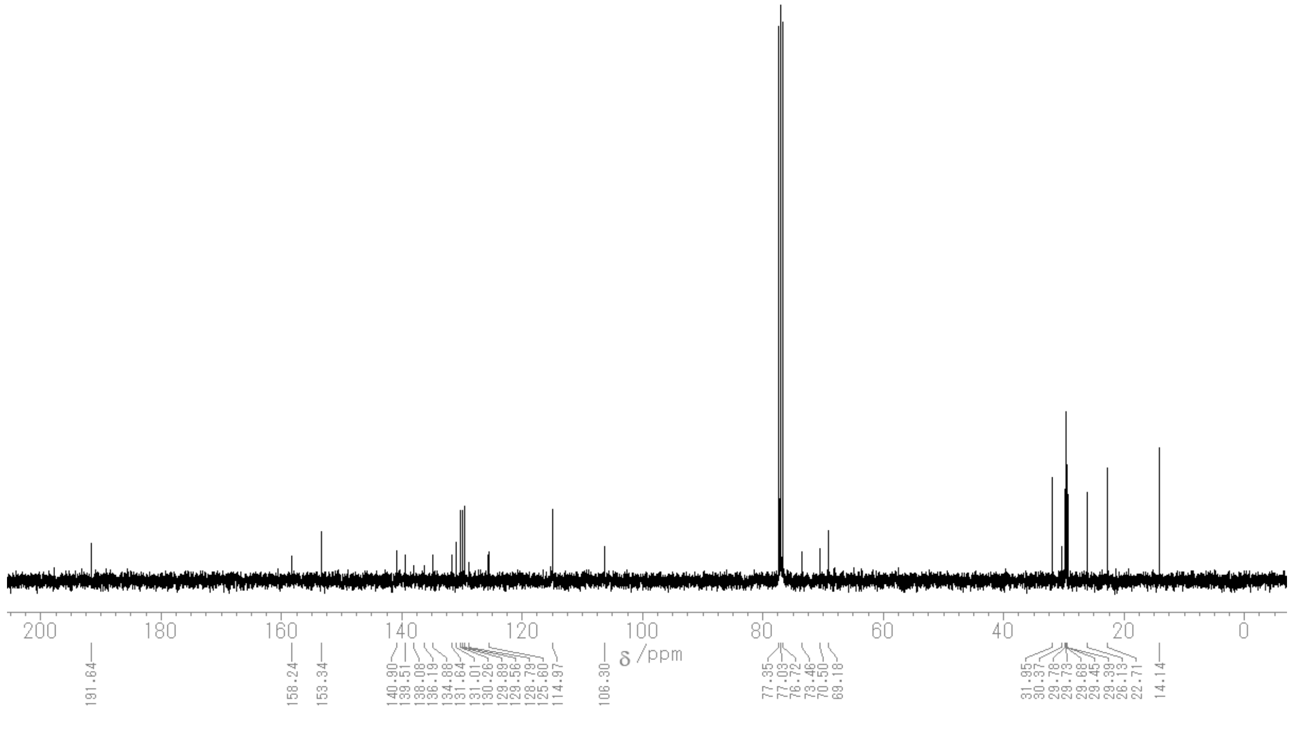

Chart S16. ^{13}C NMR spectrum of compound **9** in $CDCl_3$ at 293 K.

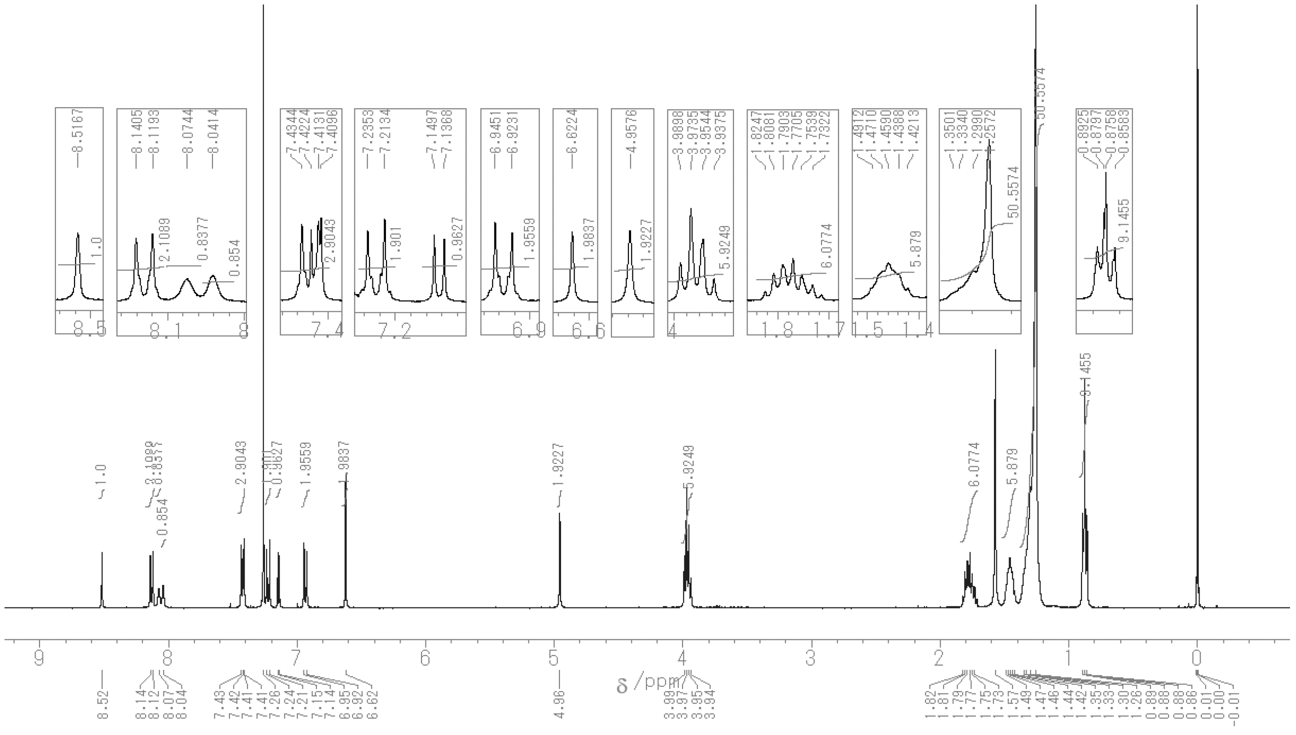

Chart S17. ^{1}H NMR spectrum of compound **2** in $CDCl_3$ at 293 K.

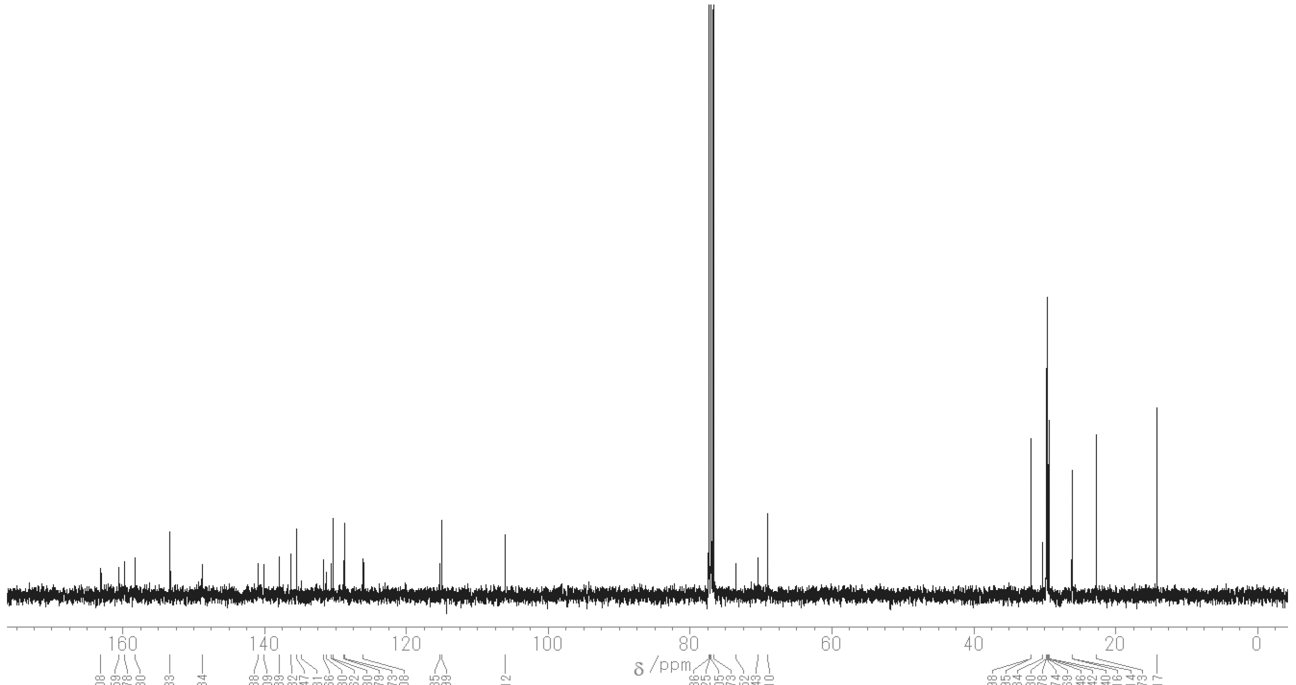

Chart S18. ^{13}C NMR spectrum of compound **2** in $CDCl_3$ at 293 K.

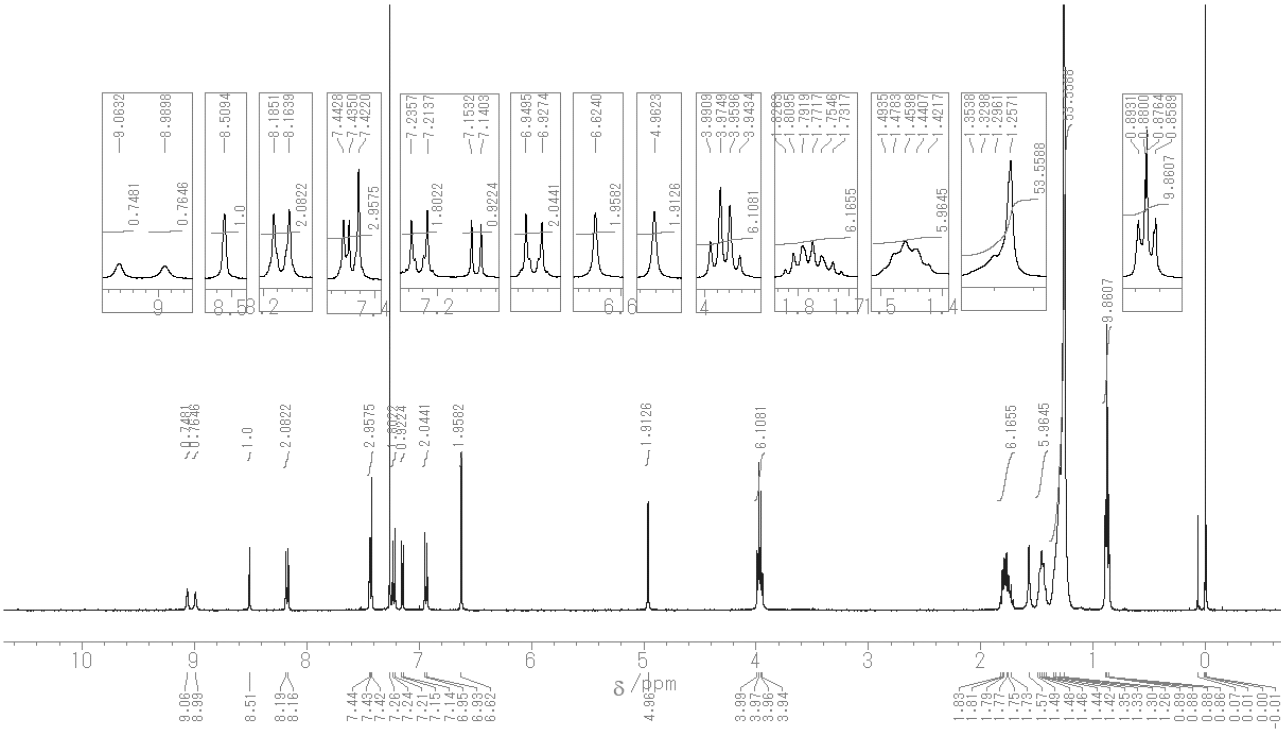

Chart S19. ^{1}H NMR spectrum of compound **2T** in $CDCl_3$ at 293 K.

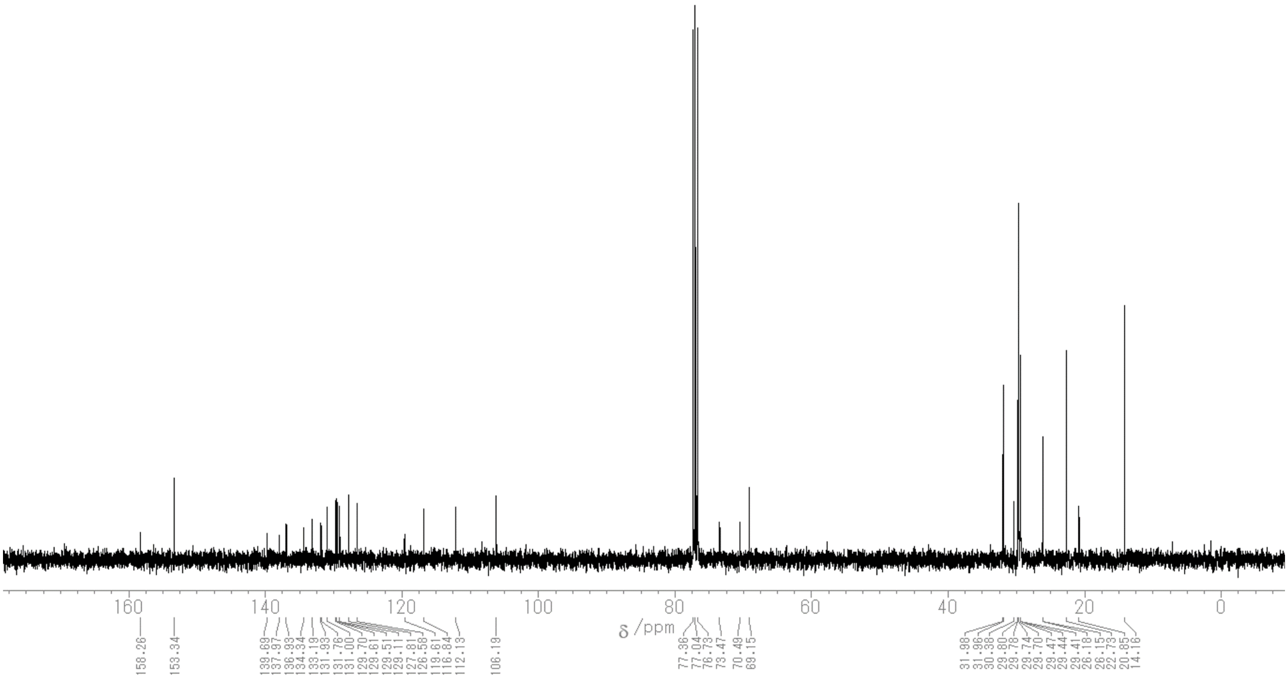

Chart S20. ^{13}C NMR spectrum of compound **2T** in $CDCl_3$ at 293 K.

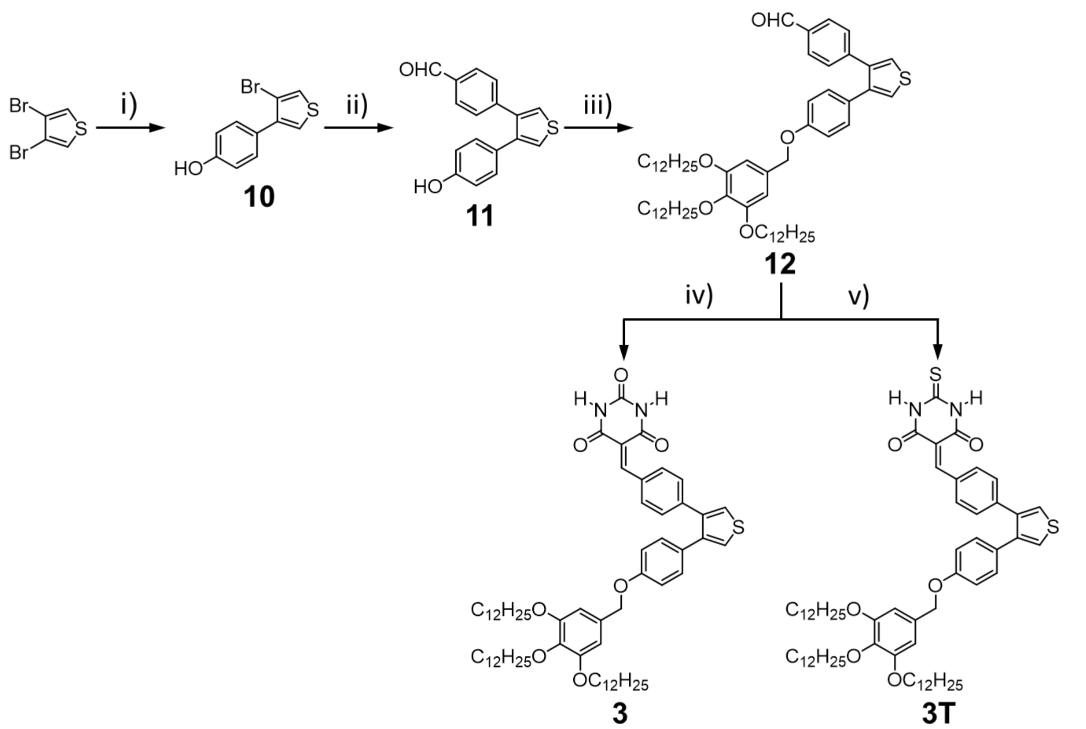

Scheme S3. Synthesis of **3** and **3T**. i) 4-hydroxyphenylboronic acid, $Pd(PPh_3)_4$, Na_2CO_3, 1,4-dioxane, 70 °C; ii) 4-formylphenylboronic acid, $Pd(PPh_3)_4$, K_2CO_3, 1,4-dioxane, 70 °C; iii) 3,4,5-tri(*n*-dodecyloxy)benzyl chloride[S4], K_2CO_3, DMF, 70 °C; iv) barbituric acid, EtOH, 70 °C, reflux; v) 2-thiobarbituric acid, EtOH, 70 °C, reflux.

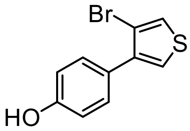

Synthesis of compound 10: 3,4-dibromothiophene (1.11 g, 4.57 mmol), 4-hydroxyphenylboronic acid (512 mg, 3.71 mmol) and $Pd(PPh_3)_4$ (65.0 mg, 0.0562 mmol) were dissolved in dry 1,4-dioxane (40 mL). To this 2.04 M aq. Na_2CO_3 (10 mL, 20.4 mmol) was added and the mixture was stirred at 70 °C for 16 h under N_2 atmosphere. After the reaction mixture was diluted with AcOEt, the resulting solution was washed with water and brine, dried over Na_2SO_4, and evaporated. The residue solid was purified by column chromatography over silica gel (AcOEt:Hexane = 1:2) to give **10** as white solids (743 mg, 78% yield). ^{1}H NMR (400 MHz, $CDCl_3$, 293 K): δ = 7.40–7.37 (d, J = 8.7 Hz, 2H), 7.35–7.34 (d, J = 3.5 Hz, 1H), 7.20–7.19 (d, J = 3.5 Hz, 1H), 6.90–6.87 (d, J = 8.7 Hz, 2H), 4.80 (s, 1H); ^{13}C NMR (100 MHz, $CDCl_3$, 293 K): δ = 155.25, 141.62, 130.46, 127.96, 123.92, 122.73, 115.15, 111.30; HRMS (APCI): *m/z* calcd for $C_{10}H_8OBrS$ 254.9474 $[M+H]^+$, found 254.9478.

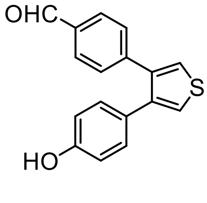

Synthesis of compound 11: Compound **10** (399 mg, 1.56 mmol), 4-formylphenylboronic acid (283 mg, 1.89 mmol) and $Pd(PPh_3)_4$ (55.0 mg, 0.0475 mmol) were dissolved in dry 1,4-dioxane (20 mL). To this 0.772 M aq. K_2CO_3 (10 mL, 7.72 mmol) was added and the mixture was stirred at 70 °C for 17 h under N_2 atmosphere. After the reaction mixture was diluted with AcOEt, the resulting

solution was washed with water and brine, dried over Na_2SO_4, and evaporated. The resulting solid was purified by column chromatography over silica gel (AcOEt:Hexane = 1:3) to give **11** as yellow solids (214 mg, 49% yield). ^{1}H NMR (400 MHz, $CDCl_3$, 293 K): δ = 9.98 (s, 1H), 7.80–7.77 (d, J = 8.5 Hz, 2H), 7.42–7.41 (d, J = 3.4 Hz, 1H), 7.37–7.35 (d, J = 8.3 Hz, 2H), 7.29–7.28 (d, J = 3.4 Hz, 1H), 7.06–7.02 (d, J = 8.7 Hz, 2H), 6.77–6.74 (d, J = 8.7 Hz, 2H), 5.16 (s, 1H); ^{13}C NMR (100 MHz, $CDCl_3$, 333 K): δ = 191.56, 155.00, 142.95, 141.42, 140.46, 135.00, 130.31, 129.53, 129.45, 128.89, 125.14, 123.79, 115.32; HRMS (APCI): *m/z* calcd for $C_{17}H_{13}O_2S$ 281.0631 $[M+H]^+$, found 281.0627.

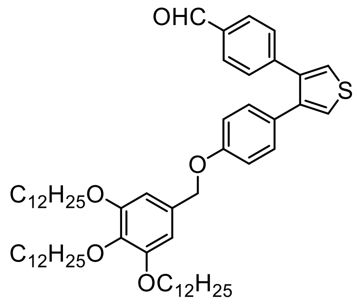

Synthesis of compound 12: Compound **11** (143 mg, 0.510 mmol), K_2CO_3 (320 mg, 2.32 mmol) and 5-(chloromethyl)-1,2,3-tris-(dodecyloxy)benzene[S4] (412 mg, 0.471 mmol) were dissolved in dry DMF (10 mL). The mixture was stirred at 70 °C for 15 h under N_2 atmosphere. After the reaction mixture was diluted with AcOEt:Hexane (1:4), the resulting solution was washed with water and brine, dried over Na_2SO_4, and evaporated. The resulting solid was purified by column chromatography over silica gel (AcOEt:Hexane = 1:4) to give **12** as pale yellow solids (250 mg, 53% yield). ^{1}H NMR (400 MHz, $CDCl_3$, 293 K): δ = 9.99 (s, 1H), 7.79–7.77 (d, J = 8.4 Hz, 2H), 7.42–7.41 (d, J = 3.3 Hz, 1H), 7.38–7.35 (d, J = 8.2 Hz, 2H), 7.30–7.28 (d, J = 3.3 Hz, 1H), 7.11–7.08 (d, J = 8.7 Hz, 2H), 6.90–6.87 (d, J = 8.7 Hz, 2H), 6.62 (s, 2H), 4.92 (s, 2H), 3.99–3.92 (m, 6H), 1.84–1.72 (m, 6H), 1.48–1.43 (m, 6H), 1.36–1.26 (m, 48H), 0.90–0.86 (m, 9H); ^{13}C NMR (100 MHz, $CDCl_3$, 293 K): δ = 191.89, 158.17, 153.34, 142.93, 141.36, 140.36, 138.08, 134.81, 131.67, 130.14, 129.68, 129.50, 128.79, 125.39, 123.92, 114.72, 106.30, 73.45, 70.48, 69.18, 31.95, 30.37, 29.78, 29.76, 29.73, 29.67, 29.45, 29.42, 29.39, 26.17, 26.13, 22.71, 14.14; HRMS (APCI): *m/z* calcd for $C_{60}H_{91}O_5S$ 923.6582 $[M+H]^+$, found 923.6581.

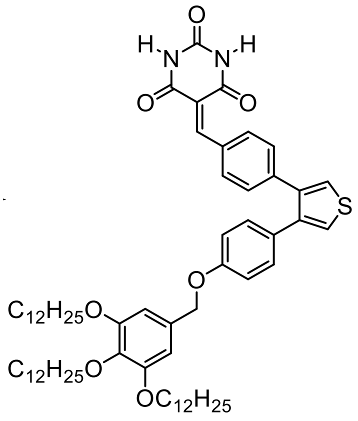

Synthesis of compound 3: Compound **12** (200 mg, 0.217 mmol) and barbituric acid (140 mg, 1.09 mmol) in EtOH (10 mL) were stirred at 70 °C for 18 h under reflux. The reaction mixture was cooled to room temperature and the resulting precipitates were collected by filtration and washed with hot ethanol repeatedly. The residue was dissolved in chloroform, and ethanol was added at 0 °C. The resulting precipitates were collected by centrifugation to give pure compound **3** as yellow solids (78 mg, 35% yield). ^{1}H NMR (400 MHz, $CDCl_3$, 293 K): δ = 8.55 (s, 1H), 8.15–8.13 (d, J = 8.5 Hz, 2H), 8.07 (s, 1H), 8.05 (s, 1H), 7.47–7.46 (d, J = 3.2 Hz,1H), 7.34–7.32 (d, J = 8.7 Hz, 2H), 7.29–7.28 (d, J = 3.3 Hz, 1H), 7.13–7.11 (d, J = 8.7 Hz, 2H), 6.91–6.89 (d, J = 8.8 Hz, 2H), 6.61 (s, 2H), 4.94 (s, 2H), 3.98–3.93 (m, 6H), 1.82–1.73 (m, 6H), 1.47–1.42

(m, 6H), 1.35–1.26 (m, 48H), 0.89–0.86 (m, 9H); ^{13}C NMR (100 MHz, $CDCl_3$, 333 K): δ = 159.85, 158.30, 153.38, 142.73, 141.48, 140.31, 138.39, 134.96, 131.79, 130.56, 130.13, 128.86, 128.83, 125.63, 123.97, 115.52, 114.90, 106.50, 73.51, 70.45, 69.37, 31.87, 30.32, 29.69, 29.63, 29.58, 29.47, 29.38, 29.31, 29.28, 26.11, 26.09, 22.60, 13.95; HRMS (ESI): *m/z* calcd for $C_{64}H_{92}O_7N_2ClS$ 1067.6308 $[M+Cl]^-$, found 1067.6338.

Synthesis of compound 3T: Compound **12** (64 mg, 0.0693 mmol) and 2-thiobarbituric acid (8.8 mg, 0.061 mmol) in EtOH (3 mL) were stirred at 70 °C for 14 h under reflux. The reaction mixture was cooled to room temperature and the resulting precipitates were collected by filtration and washed with hot methanol repeatedly to give pure compound **3T** as orange solids (61 mg, 84% yield). ^{1}H NMR (400 MHz, $CDCl_3$, 293 K): δ = 9.06 (s, 1H), 8.97 (s, 1H), 8.54 (s, 1H), 8.19–8.17 (d, J = 8.4 Hz, 2H), 7.48–7.47 (d, J = 3.3 Hz, 1H), 7.35–7.33 (d, J = 8.4 Hz, 2H), 7.30–7.29 (d, J = 3.3 Hz, 1H), 7.13–7.11 (d, J = 8.5 Hz, 2H), 6.91–6.89 (d, J = 8.8 Hz, 2H), 6.61 (s, 2H), 4.94 (s, 2H), 3.98–3.94 (m, 6H), 1.81–1.73 (m, 6H), 1.47–1.42 (m, 6H), 1.35–1.26 (m, 48H), 0.89–0.86 (m, 9H); ^{13}C NMR (100 MHz, $CDCl_3$, 293 K): δ = 175.81, 161.31, 160.72, 158.67, 158.17, 153.32, 143.31, 141.39, 140.16, 137.87, 135.69, 131.69, 130.57, 128.97, 128.67, 126.07, 124.17, 115.27, 114.75, 106.09, 73.53, 70.39, 69.10, 31.98, 31.96, 30.34, 29.80, 29.74, 29.69, 29.46, 29.43, 29.41, 26.15, 22.73, 14.17; HRMS (APCI): *m/z* calcd for $C_{64}H_{93}O_6N_2S_2$ 1049.6470 $[M+H]^+$, found 1049.6473.

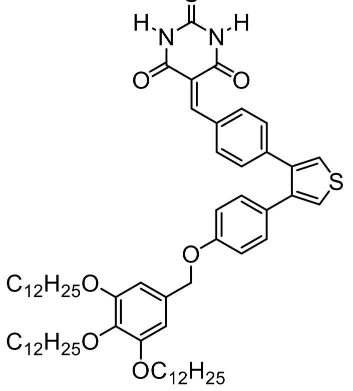

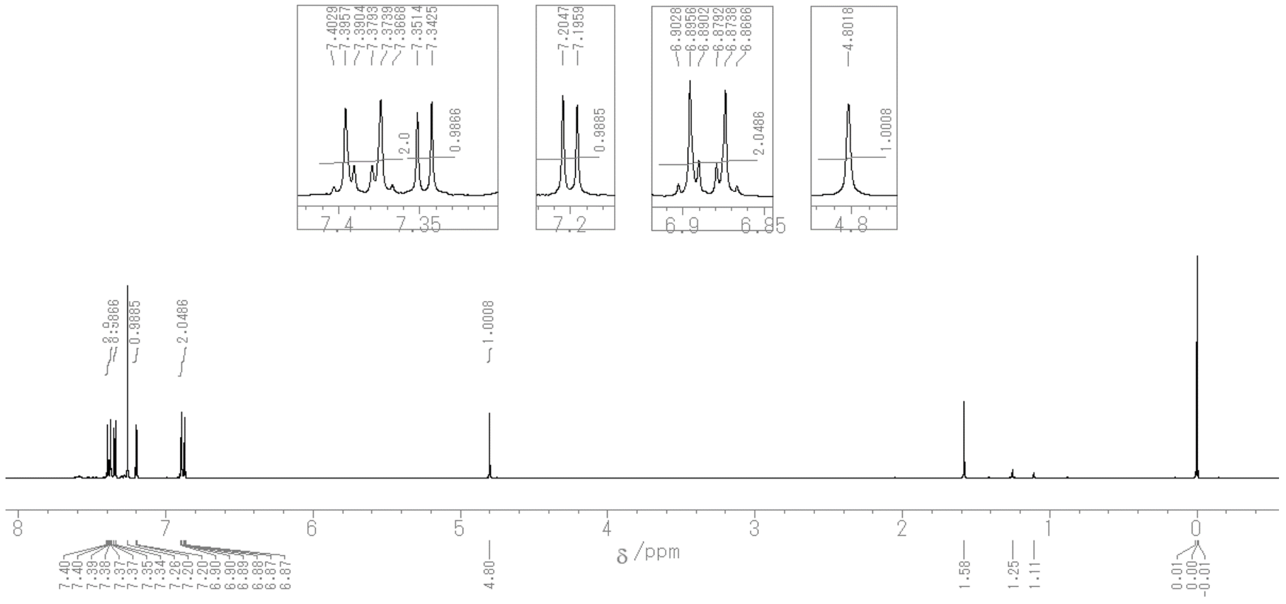

Chart S21. ^{1}H NMR spectrum of compound **10** in $CDCl_3$ at 293 K.

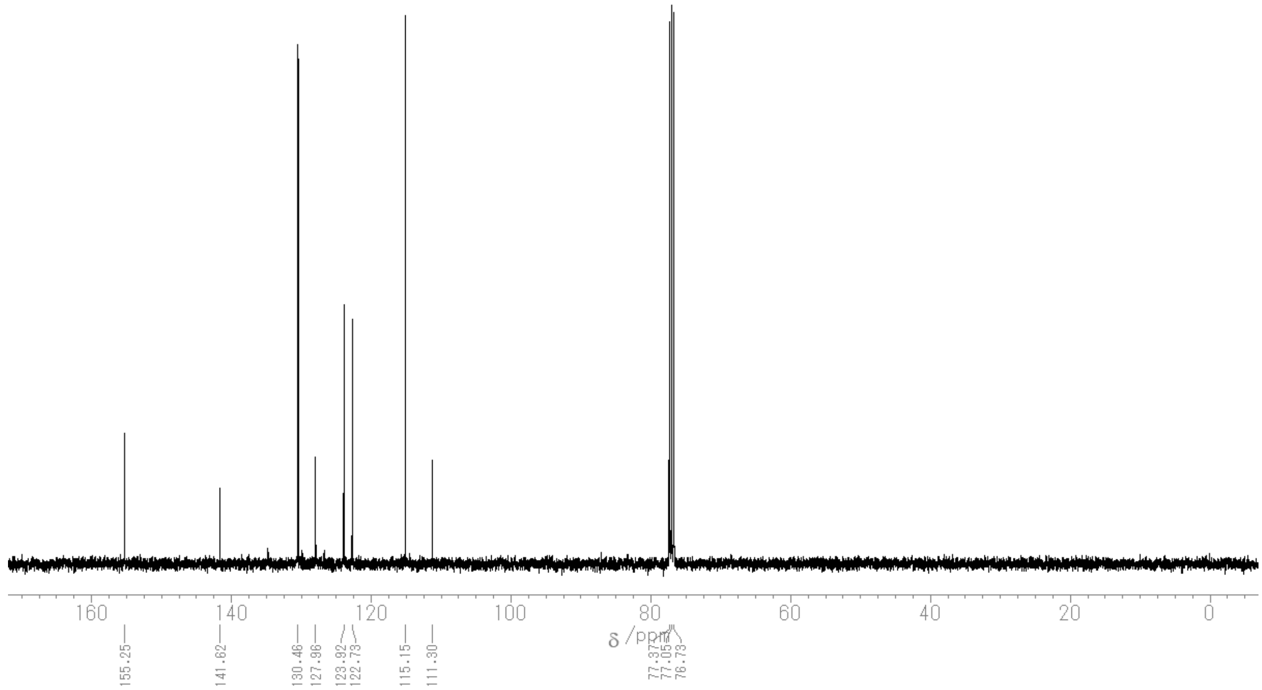

Chart S22. ^{13}C NMR spectrum of compound **10** in $CDCl_3$ at 293 K.

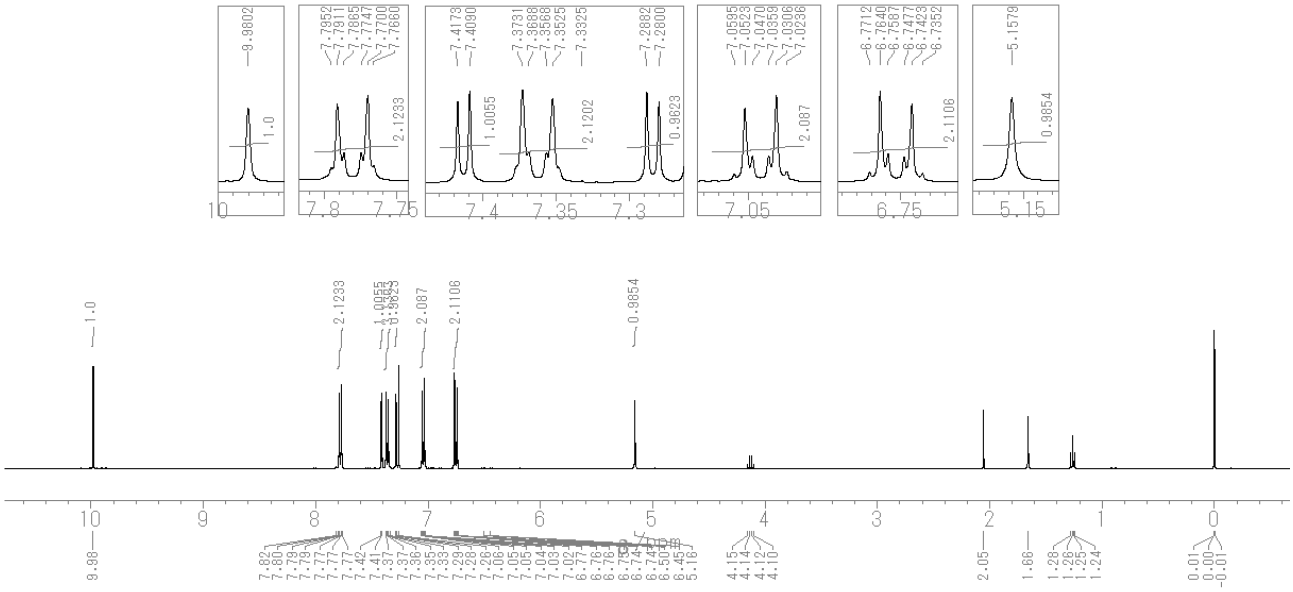

Chart S23. ^{1}H NMR spectrum of compound **11** in $CDCl_3$ at 293 K.

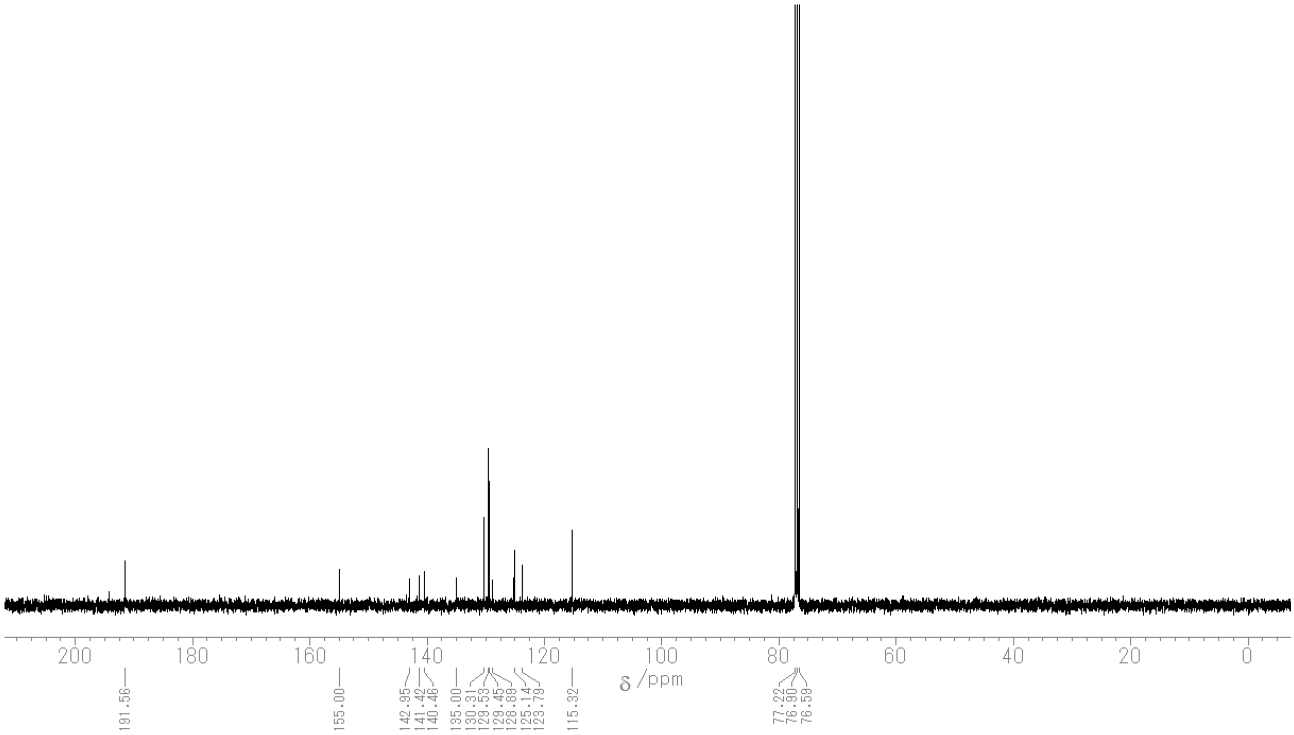

Chart S24. ^{13}C NMR spectrum of compound **11** in $CDCl_3$ at 333 K.

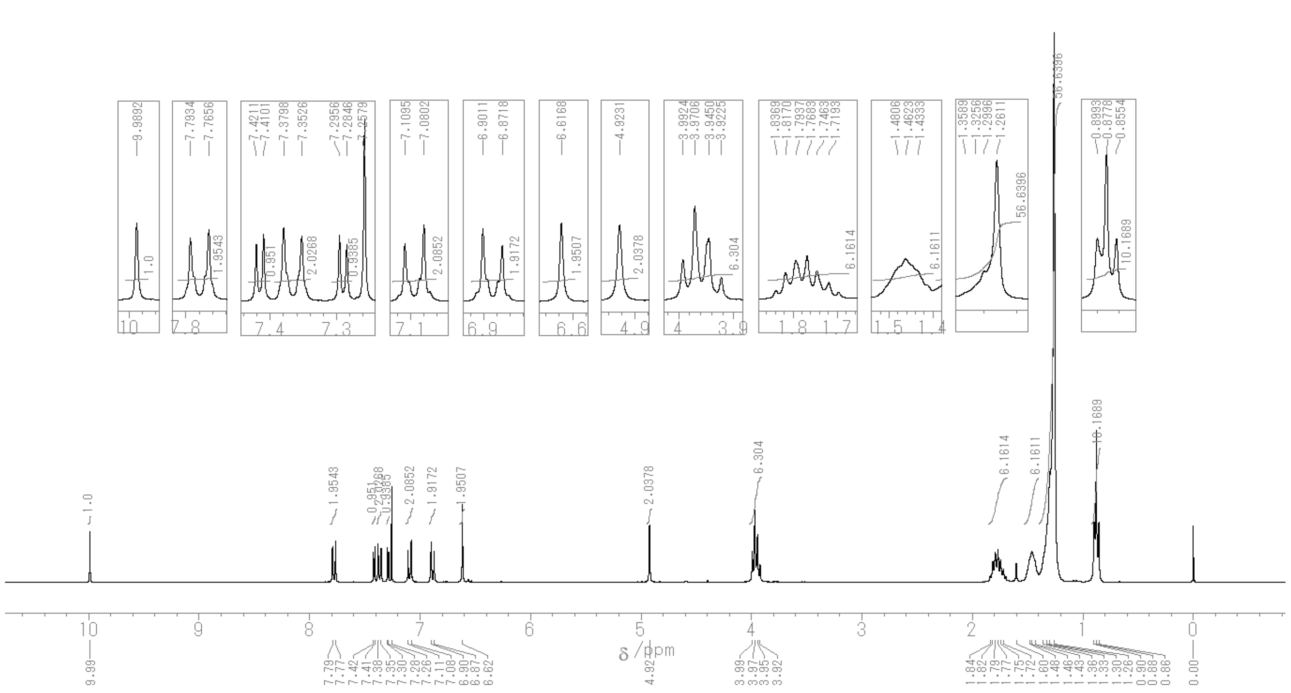

Chart S25. ^{1}H NMR spectrum of compound **12** in $CDCl_3$ at 293 K.

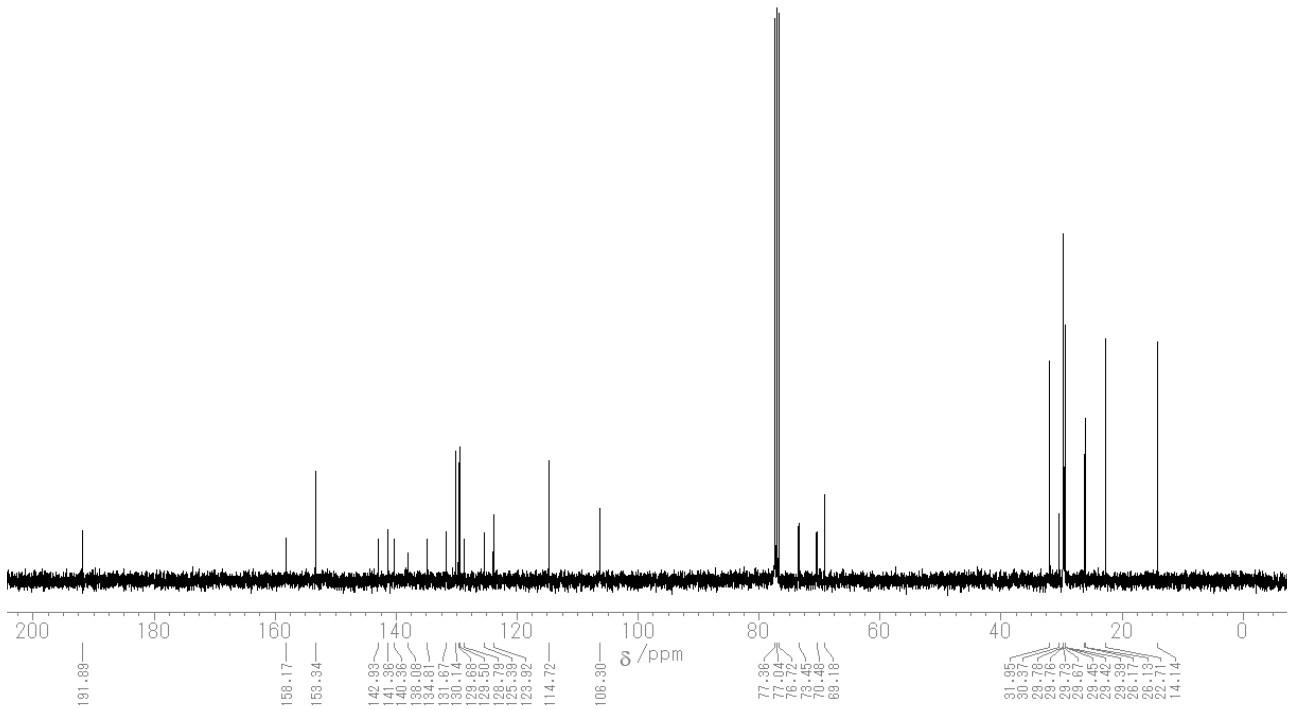

Chart S26. ^{13}C NMR spectrum of compound **12** in $CDCl_3$ at 293 K.

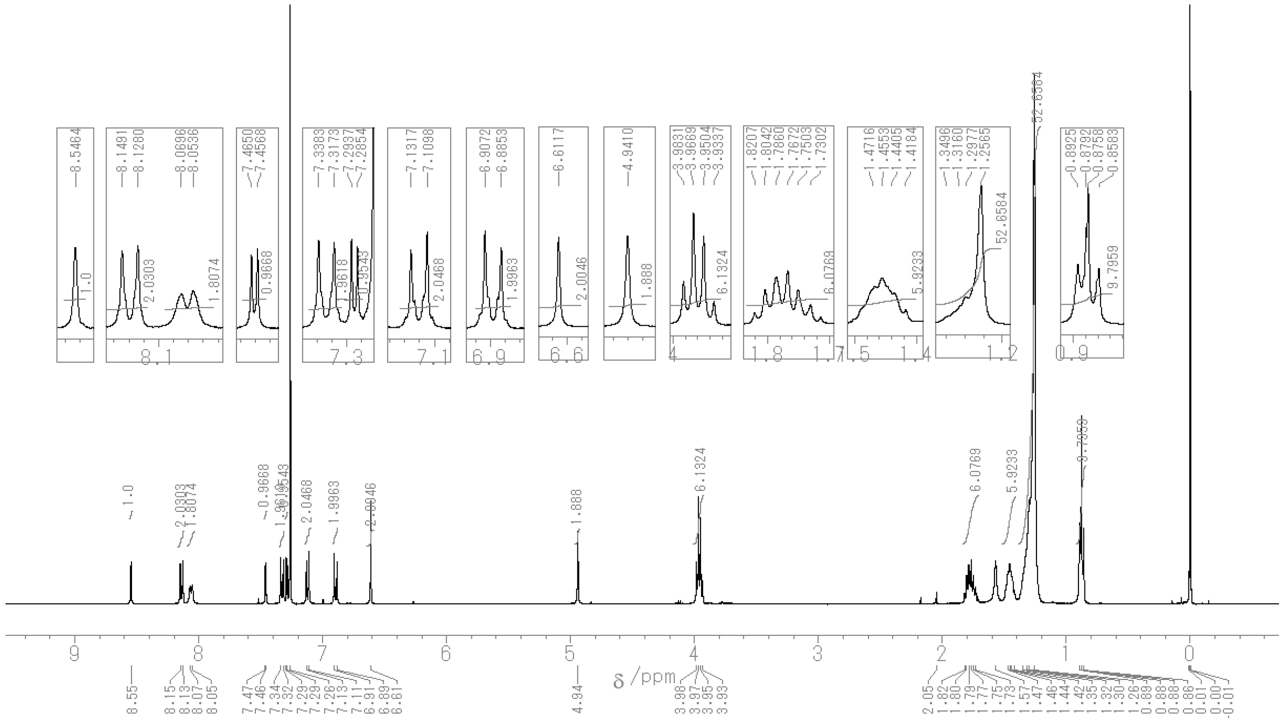

Chart S27. ^{1}H NMR spectrum of compound **3** in $CDCl_3$ at 293 K.

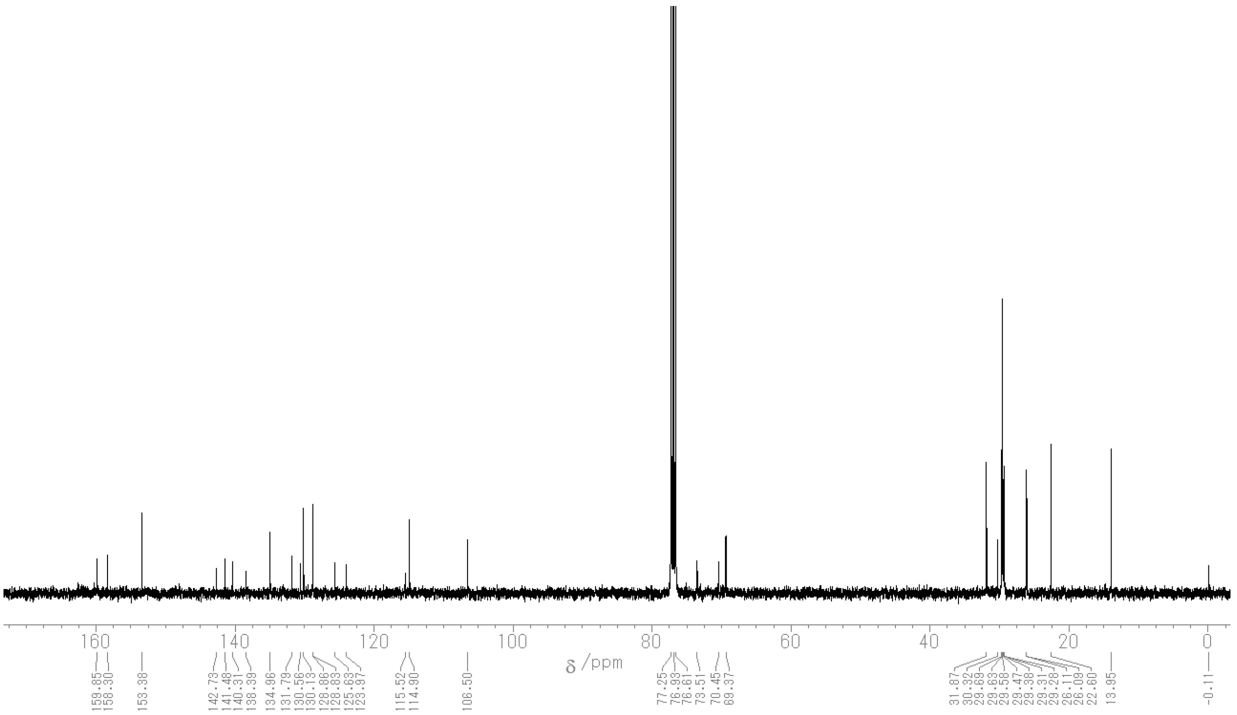

Chart S28. ^{13}C NMR spectrum of compound **3** in $CDCl_3$ at 333 K.

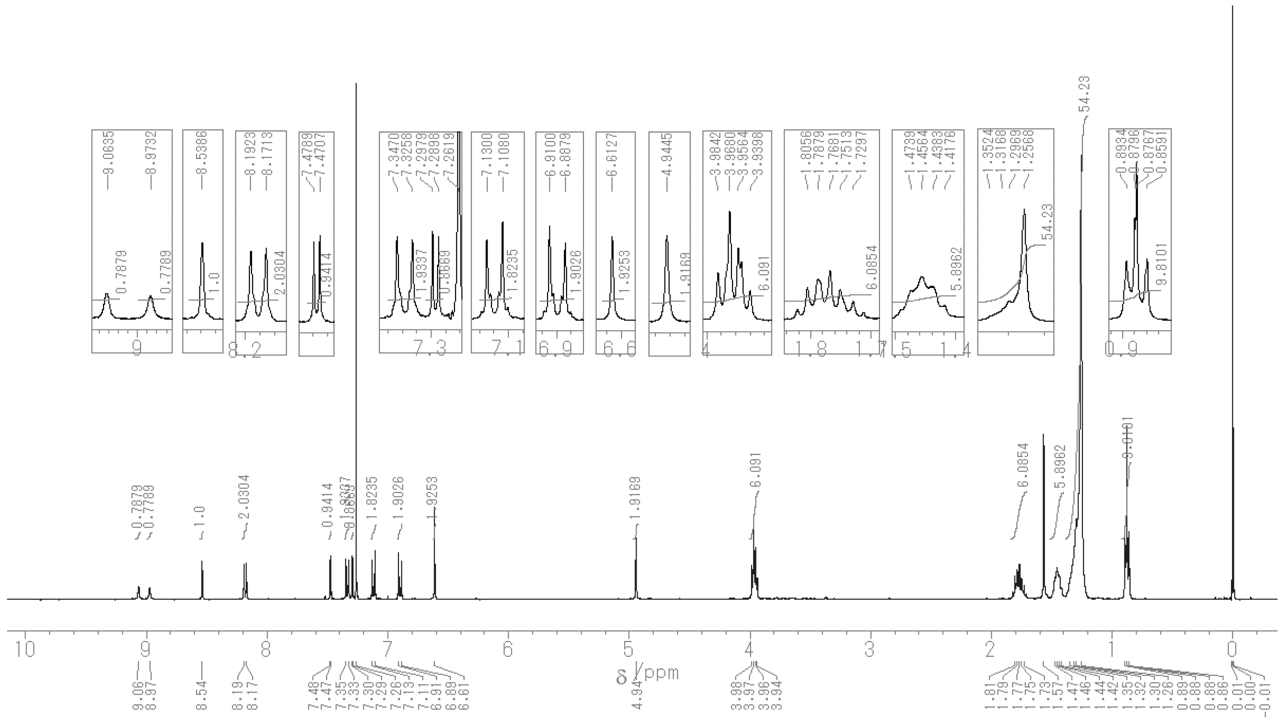

Chart S29. ^{1}H NMR spectrum of compound **3T** in $CDCl_3$ at 293 K.

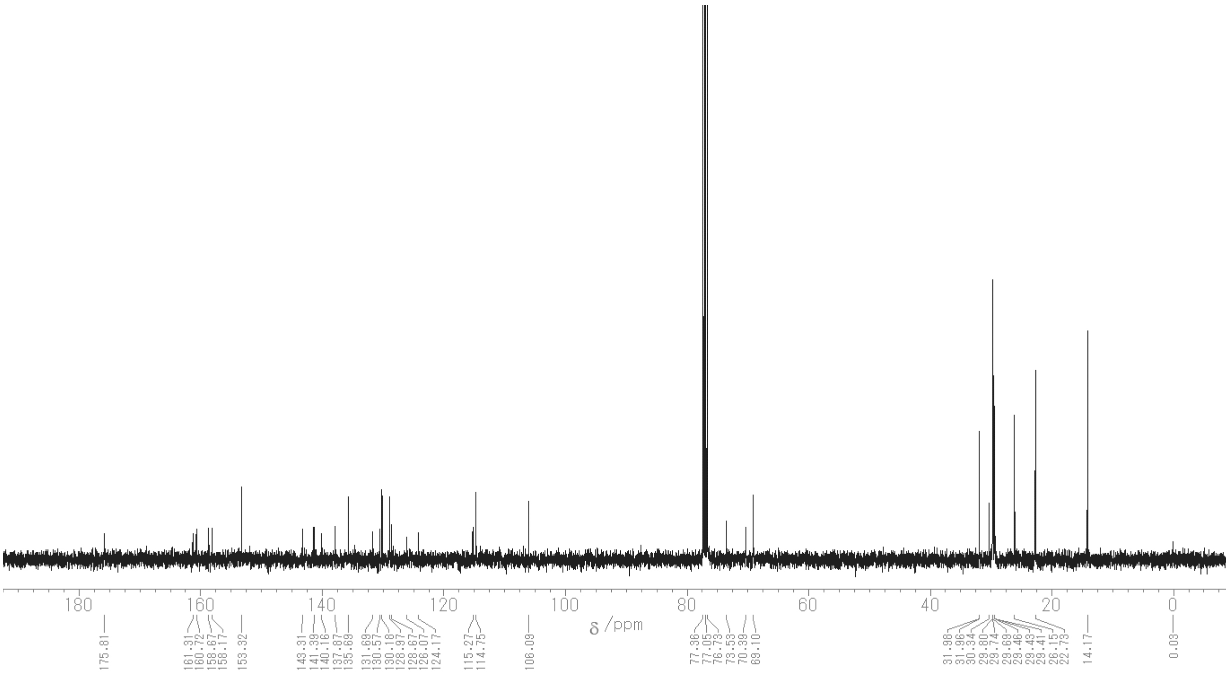

Chart S30. ^{13}C NMR spectrum of compound **3T** in $CDCl_3$ at 293 K.

3. Supporting Table

Table S1. Fluorescence properties of **1–3** in $CHCl_3$ (c = 100 μM) and in 90:10 MCH/$CHCl_3$ (v/v) solution (c = 100 μM).

Compound	Φ_{FL}	τ_{FL} (ns)	k_r (s^{-1})	k_{nr} (s^{-1})
**1** ($CHCl_3$ sol.)	0.006	0.095[a]	6.3×10^7	1.0×10^{11}
**1** (90% MCH sol.)	0.24	3.25	7.4×10^7	2.3×10^8
**2** ($CHCl_3$ sol.)	0.008	0.083[a]	9.6×10^7	1.2×10^{11}
**2** (90% MCH sol.)	0.44	3.09	1.4×10^8	1.8×10^8
**3** ($CHCl_3$ sol.)	0.003	0.032[a]	9.3×10^7	3.1×10^{11}
**3** (90% MCH sol.)	0.04	1.94	2.1×10^7	4.9×10^8

[a] Stray light error cannot be ignored due to weak emission.

4. Supporting Figures

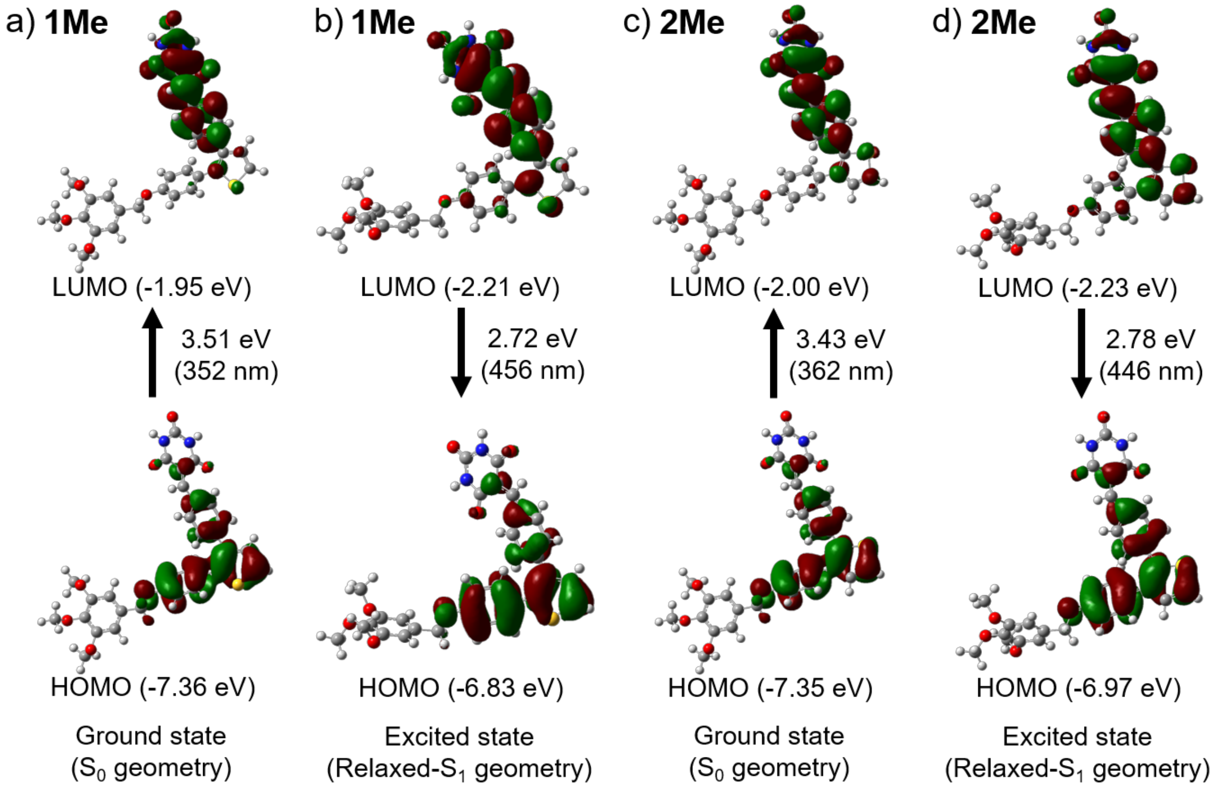

Fig. S1. Kohn–Sham orbitals for (a,c) the ground-state and (b,d) the relaxed excited-state geometries of (a,b) **1Me** and (c,d) **2Me** calculated at the CAM-B3LYP/6-31+G(d,p) level. The orbitals most contributed to the lowest-energy electronic transition are shown together with the corresponding transition energies calculated by TD-DFT at the same level. For all calculations, dodecyl groups were replaced with methyl groups.

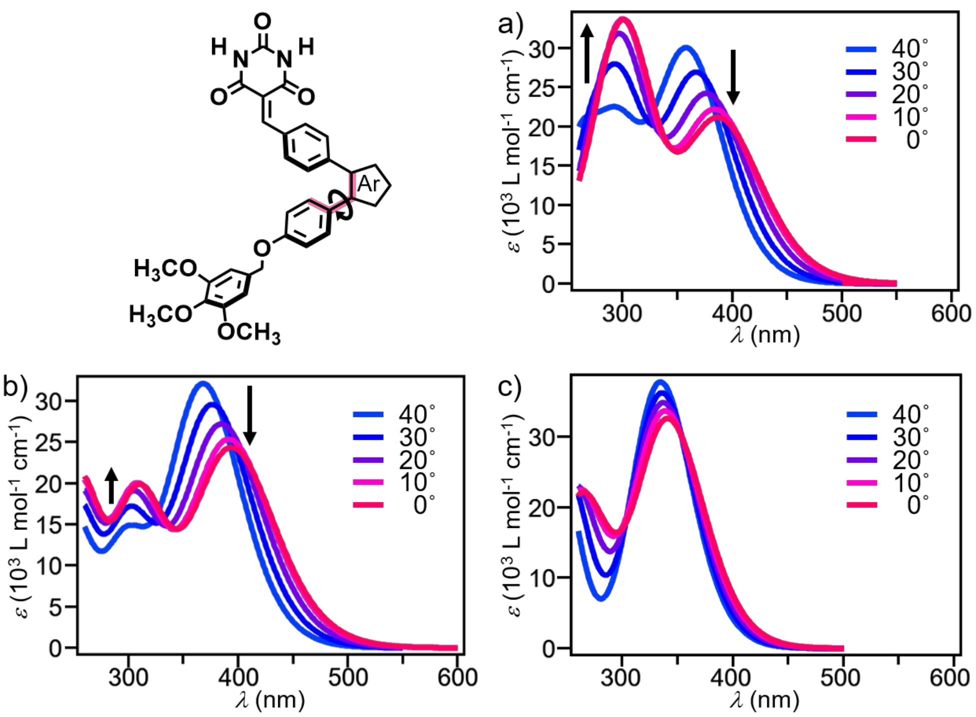

Fig. S2. Calculated absorption spactra (TD-CAM-B3LYP/6-31+G(d,p) level of theory) of (a) **1Me** (b) **2Me** and (c) **3Me** upon changing the dihedral angle from 40 to 0° between the thiophene and the benzyloxy-substituted benzene. For all calculations, dodecyl groups were replaced with methyl groups.

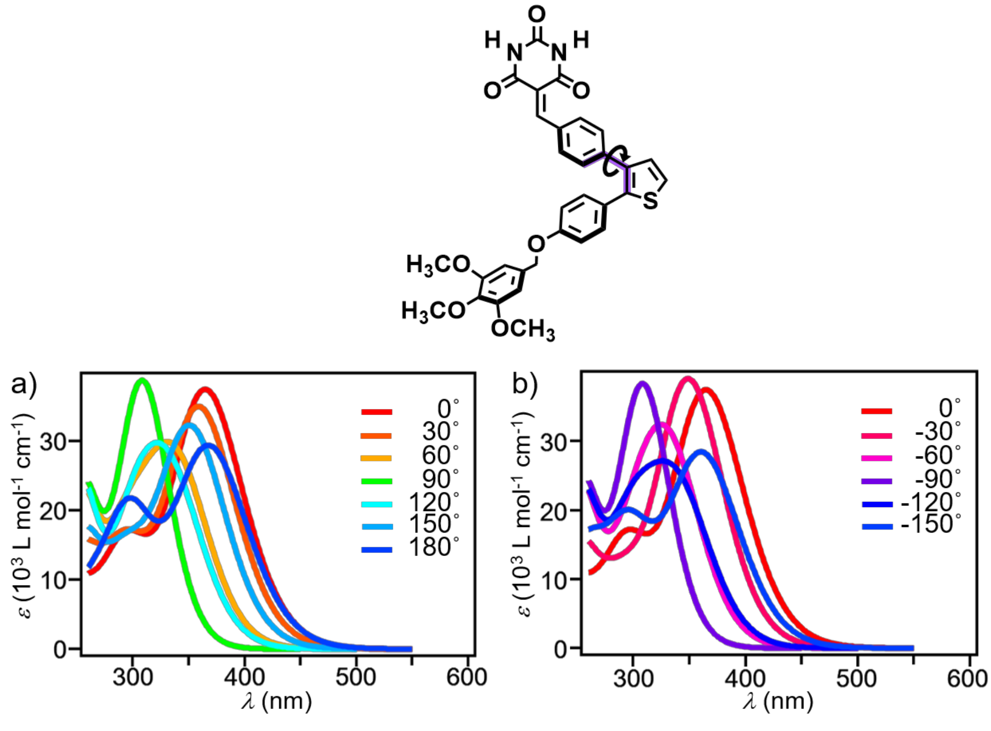

Fig. S3. Calculated absorption spactra (TD-CAM-B3LYP/6-31+G(d,p) level of theory) of **1Me** upon changing the dihedral angle from (a) 0 to 180° and (b) 0 to -150°, respectively,

between the barbiturated benzene and the thiophene. For all calculations, dodecyl groups were replaced with methyl groups.

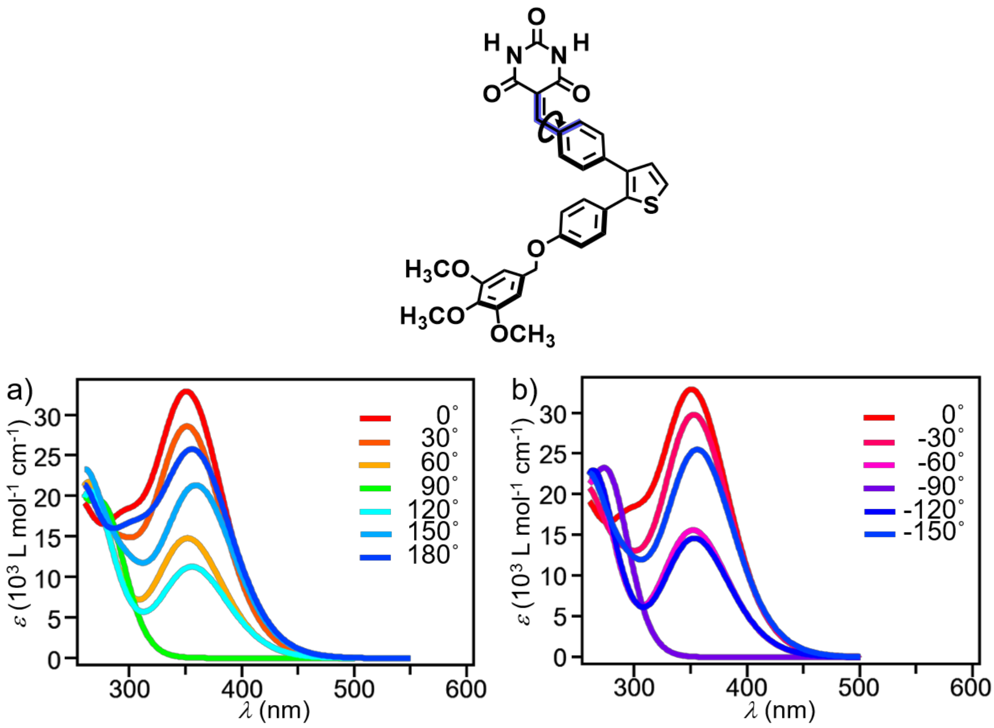

Fig. S4. Calculated absorption spactra (TD-CAM-B3LYP/6-31+G(d,p) level of theory) of **1Me** upon changing the dihedral angle from (a) 0 to 180° and (b) 0 to -150°, respectively, between the barbiturate unit and the benzene ring of the benzilidene unit. For all calculations, dodecyl groups were replaced with methyl groups.

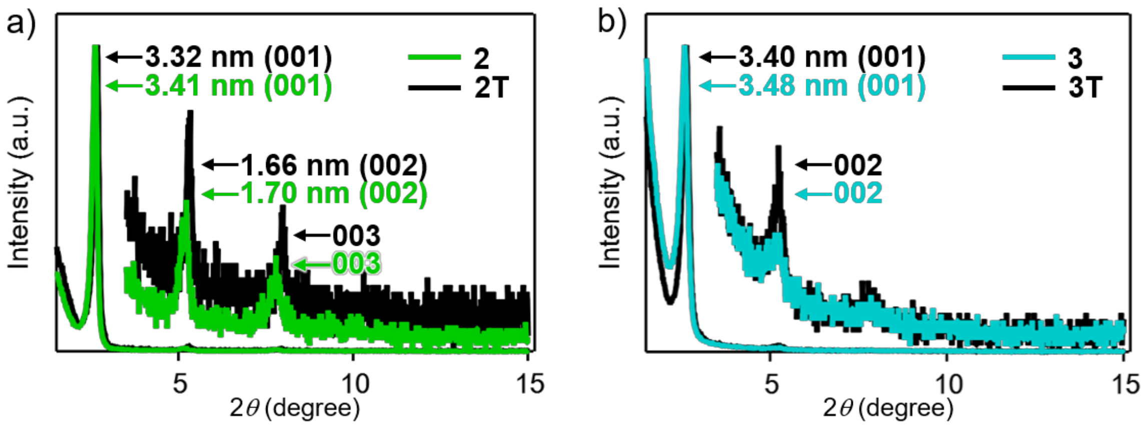

Fig. S5. Powder XRD patterns of (a) **2** and **2T** and (b) **3** and **3T**. All the powder samples were obtained by aging 90:10 MCH/$CHCl_3$ solutions (c = 100 μM) at room temperature.

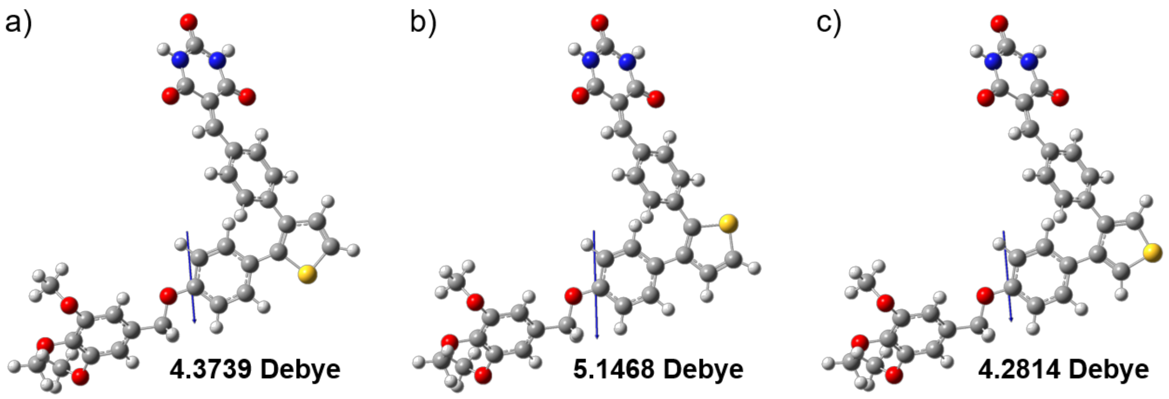

Fig. S6. Calculated electric dipole moment (CAM-B3LYP/6-31+G(d,p) level of theory) of (a) **1Me** (b) **2Me** and (c) **3Me**. For all calculations, dodecyl groups were replaced with methyl groups.

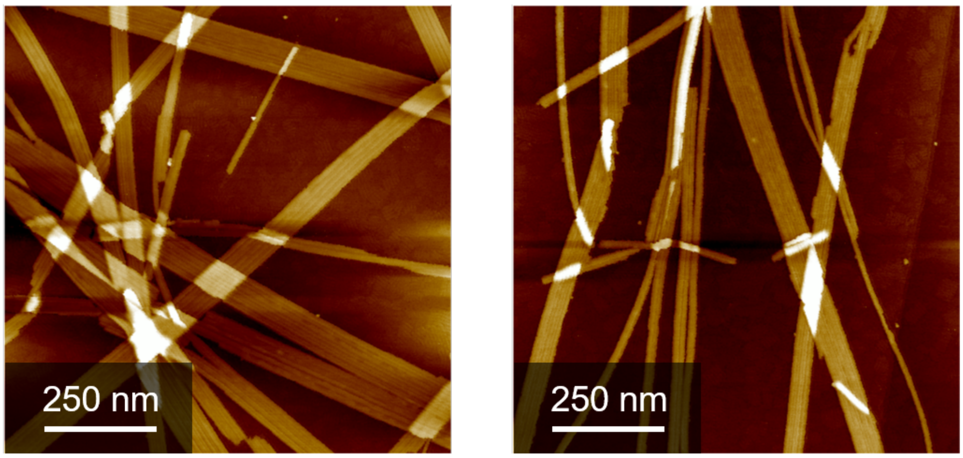

Fig. S7. AFM images of **1** in 90:10 MCH/$CHCl_3$ mixture (c = 100 μM) after aging for 15 h without stirring.

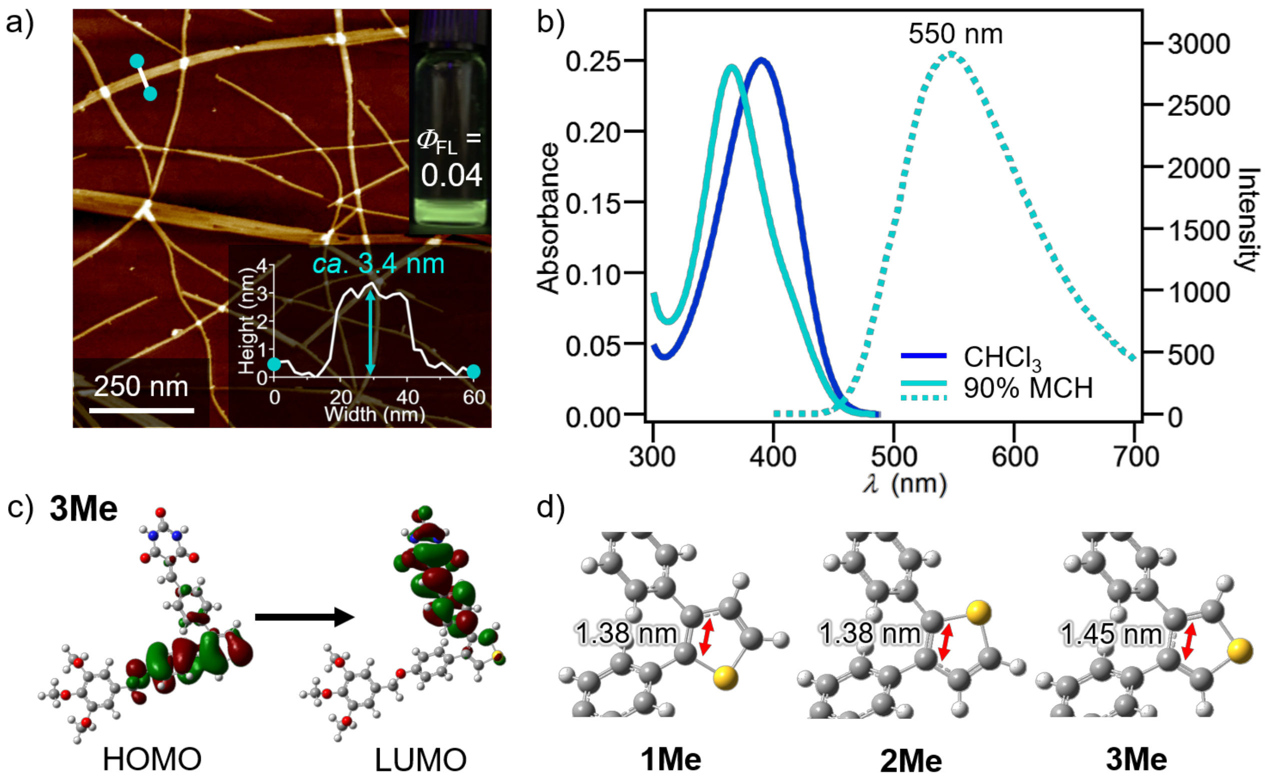

Fig. S8. (a) AFM image of supramolecular polymers of **3** formed in 90:10 MCH/$CHCl_3$ (v/v) mixture (c = 100 μM). Insets show a photograph of the corresponding solution under 365-nm light illumination and cross-sectional analysis between blue dots in the AFM image. (b) UV/vis absorption spectra (left axis) and fluorescence spectrum (right axis, excited at 365 nm) of **3** in $CHCl_3$ and 90:10 MCH/$CHCl_3$ mixture (c = 100 μM). (c) Molecular orbitals of **3Me** corresponding to the $S_0 \rightarrow S_1$ transition, calculated at the TD-CAM-B3LYP/6-31+G(d,p) level of theory. The molecular structure of **3Me** was optimized by DFT calculation at the same level of theory. For all calculations, the dodecyl groups were replaced with methyl groups. (d) Partial structures of **1Me**–**3Me** at the S_0 state (calculated at the CAM-B3LYP/6-31G+(d,p) level) showing the bond length between the two carbons of the thiophene ring substituted with phenylene groups. For all calculations, dodecyl groups were replaced with methyl groups.

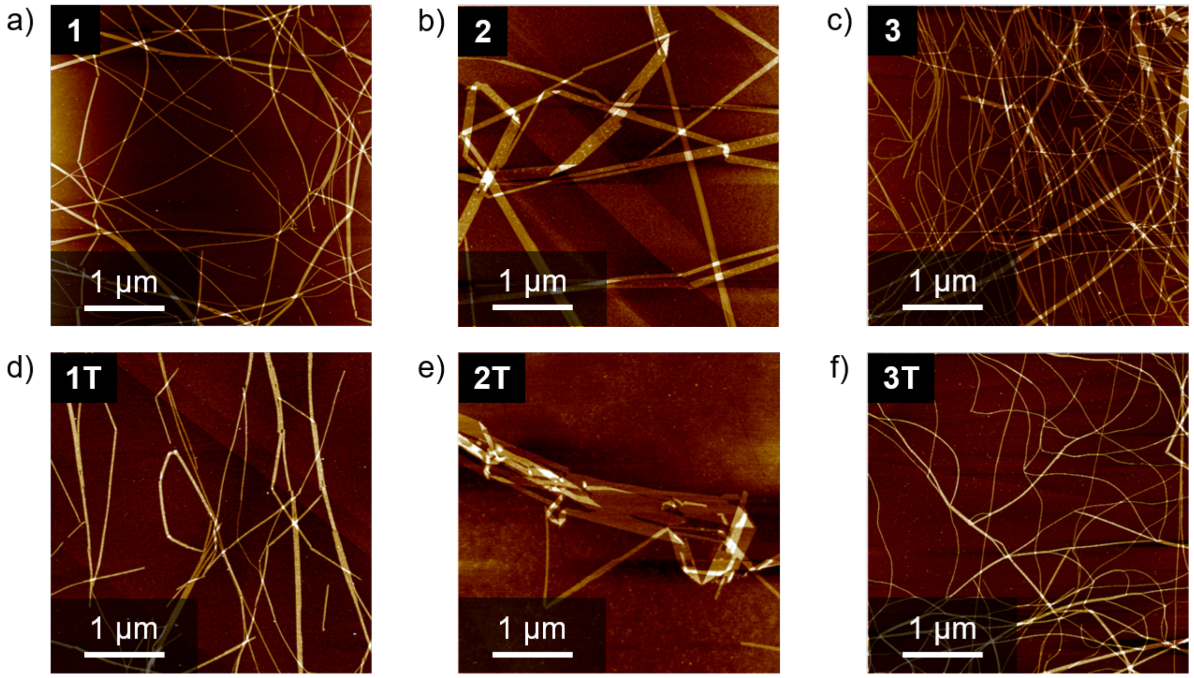

Fig. S9. AFM images of supramolecular polymers of (a) **1**, (b) **2**, (c) **3**, (d) **1T**, (e) **2T** and (f) **3T** formed in 90:10 MCH/$CHCl_3$ mixture (c = 100 μM).

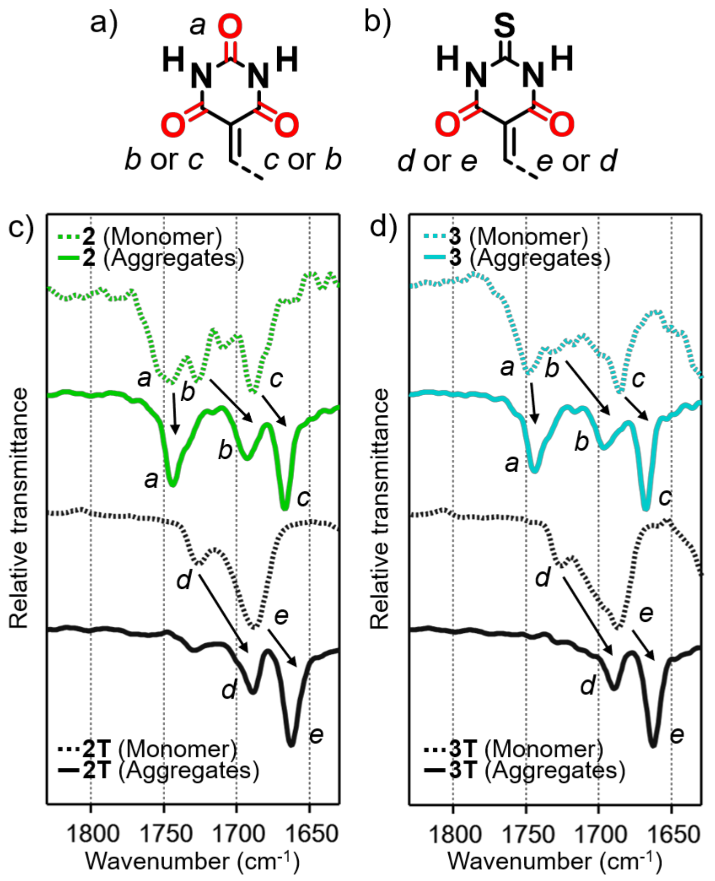

Fig. S10. (a,b) Molecular structures of (a) barbiturate and (b) 2-thiobarbiturate moieties. (c,d) FT-IR spectra (C=O stretching bands) of (c) **2** (green lines) and **2T**(black lines) and (d) **3** (blue

lines) and **3T** (black lines) in monomeric (in $CHCl_3$, dashed lines) and aggregated states (in 90:10 MCH/$CHCl_3$ mixture, solid lines). c = 100 μM for all solutions.

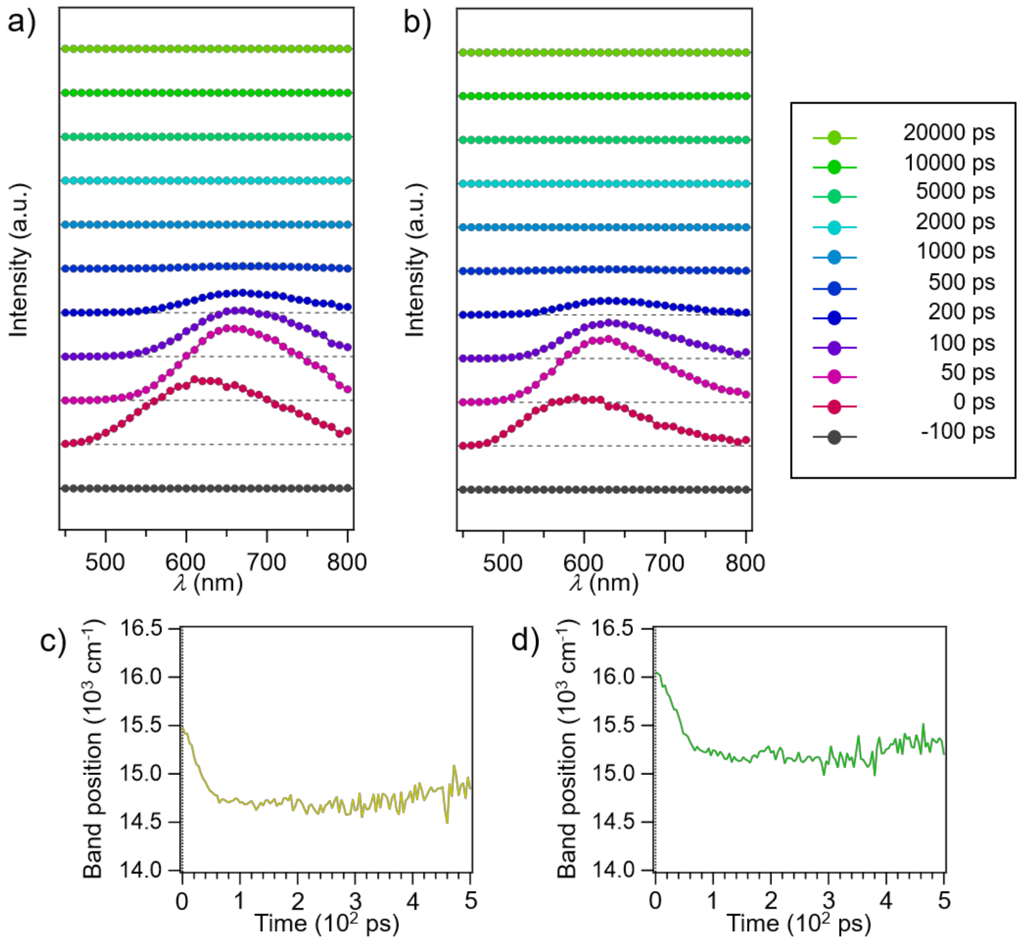

Fig. S11. (a,b) Time-resolved fluorescence spectra of monomeric (a) **1** and (b) **2** in $CHCl_3$ (c = 100 μM). **1** and **2** were excited at 430 nm. (c,d) Time evolutions of the fluorescence band position (center of mass) of (c) **1** and (d) **2**.

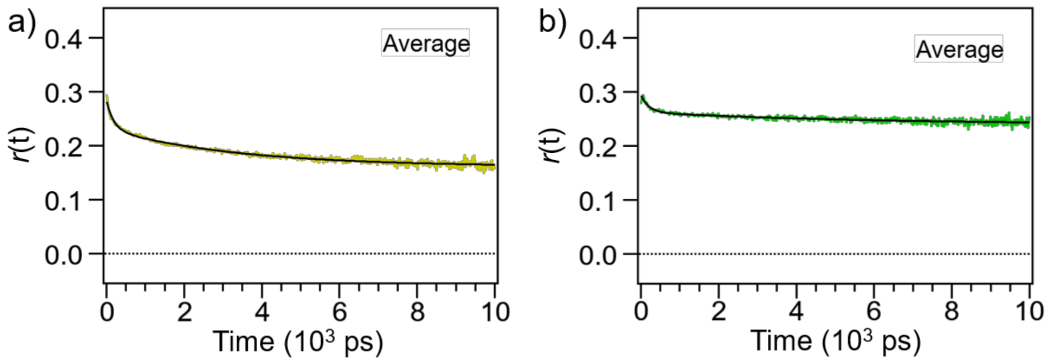

Fig. S12. Time profiles of fluorescence anisotropy of aggregated (a) **1** and (b) **2** in 90:10 MCH/$CHCl_3$ mixture (c = 100 μM). The monitoring wavelengths were respectively set to 580

and 560 nm. The data acquisitions were repeated three times for checking stability of the fluorescence signals. Black line is bi-exponential fit.

5. Supporting References

S1. M. J. Frisch, G. W. Trucks, H. B. Schlegel, G. E. Scuseria, M. A. Robb, J. R. Cheeseman, G. Scalmani, V. Barone, G. A. Petersson, H. Nakatsuji, X. Li, M. Caricato, A. V. Marenich, J. Bloino, B. G. Janesko, R. Gomperts, B. Mennucci, H. P. Hratchian, J. V. Ortiz, A. F. Izmaylov, J. L. Sonnenberg, D. Williams−Young, F. Ding, F. Lipparini, F. Egidi, J. Goings, B. Peng, A. Petrone, T. Henderson, D. Ranasinghe, V. G. Zakrzewski, J. Gao, N. Rega, G. Zheng, W. Liang, M. Hada, M. Ehara, K. Toyota, R. Fukuda, J. Hasegawa, M. Ishida, T. Nakajima, Y. Honda, O. Kitao, H. Nakai, T. Vreven, K. Throssell, J. A. Montgomery, Jr., J. E. Peralta, F. Ogliaro, M. J. Bearpark, J. J. Heyd, E. N. Brothers, K. N. Kudin, V. N. Staroverov, T. A. Keith, R. Kobayashi, J. Normand, K. Raghavachari, A. P. Rendell, J. C. Burant, S. S. Iyengar, J. Tomasi, M. Cossi, J. M. Millam, M. Klene, C. Adamo, R. Cammi, J. W. Ochterski, R. L. Martin, K. Morokuma, O. Farkas, J. B. Foresman and D. J. Fox, *Gaussian 16* (*Revision B.01*), Wallingford CT, 2016.

S2. N. Boens, W. Qin, N. Basaric, J. Hofkens, M. Ameloot, J. Pouget, J. P. Lefevre, B. Valeur, E. Gratton, M. Vandeven, N. D. Silva, Y. Engelborghs, K. Willaert, A. Sillen, G. Rumbles, D. Phillips, A. Visser, A. van Hoek, J. R. Lakowicz, H. Malak, I. Gryczynski, A. G. Szabo, D. T. Krajcarski, N. Tamai and A. Miura, *Anal. Chem.*, 2007, **79**, 2137–2149.

S3. J. R. Lakowicz, *Principles of Fluorescence Spectroscopy*, 3rd ed.; Springer-Verlag: New York, 2006.

S4. V. S. K. Balagurusamy, G. Ungar, V. Percec and G. Johansson, *J. Am. Chem. Soc.*, 1997, **119**, 1539–1555.
